# Identification of berberine as a novel drug for the treatment of multiple myeloma via targeting UHRF1

**DOI:** 10.1186/s12915-020-00766-8

**Published:** 2020-03-25

**Authors:** Chunming Gu, Zhao Yin, Hong Nie, Yanjun Liu, Juhua Yang, Guiping Huang, Jianping Shen, Liguo Chen, Jia Fei

**Affiliations:** 1 Department of Biochemistry and Molecular Biology, Medical College of Jinan University, 601 Western Huangpu Avenue, Guangzhou, 510632 China; 2 Institute of Chinese Integrative Medicine, Chinese Medicine College, Jinan University, Guangzhou, 510632 China; 3 Guangdong Province Key Laboratory of Pharmacodynamic Constituents of TCM and New Drugs Research, College of Pharmacy, Jinan University, Guangzhou, 510632 China; 4 International Cooperative Laboratory of Traditional Chinese Medicine Modernization and Innovative Drug Development of Chinese Ministry of Education (MOE), College of Pharmacy, Jinan University, Guangzhou, 510632 China; 5 Department of Hematology, The First Affiliated Hospital of Zhejiang Chinese Medical University, Hangzhou, 310006 China

**Keywords:** Berberine, Multiple myeloma, SPR-LC-MS/MS approach, UHRF1, Target identification

## Abstract

**Background:**

Current therapies for multiple myeloma (MM) are associated with toxicity and resistance, highlighting the need for novel effective therapeutics. Berberine (BBR), a botanical alkaloid derived from several Berberis medicinal plants, has exhibited anti-tumor effects, including against multiple myeloma (MM); however, the molecular mechanism underlying the anti-MM effect has not been previously described. This study aimed to identify the target of berberine and related mechanisms involved in its therapeutic activity against MM.

**Results:**

Here, we demonstrated that BBR treatment killed MM cells in vitro and prolonged the survival of mice bearing MM xenografts in vivo. A screening approach integrating surface plasmon resonance (SPR) with liquid chromatography-tandem mass spectrometry (LC-MS/MS) identified UHRF1 (ubiquitin-like with PHD and RING Finger domains 1) as a potential target of BBR. Combining molecular docking and SPR analysis, we confirmed UHRF1 as a BBR-binding protein and discovered that BBR binds UHRF1 in the tandem tudor domain and plant homeodomain (TTD-PHD domain). BBR treatment induced UHRF1 degradation via the ubiquitin-dependent proteasome system and reactivated p16^INK4A^ and p73 in MM cells. Overexpression of UHRF1 promoted the MM cell proliferation and rendered MM cells more resistant to BBR, while silencing of UHRF1 with siRNA attenuated BBR-induced cytotoxicity.

**Conclusions:**

In summary, our study has identified UHRF1 as a direct target of BBR and uncovered molecular mechanisms involved in the anti-MM activity of BBR. Targeting UHRF1 through BBR may be a novel therapeutic strategy against MM.

## Background

Multiple myeloma (MM) is an incurable malignant hematological disease, characterized by the abnormal proliferation of clonal plasma cells in the bone marrow [1]. MM ranks the second in terms of incidence among hematologic malignancies, with a 5-year survival rate of < 50% [2]. Bortezomib has revolutionized MM therapies in the previous decades [3]. Bortezomib targets the 26S proteasome subunit β5 and exerts anti-MM effects by inhibiting the secretion of interleukin-6 (IL-6) through the NF-κB signaling pathway. It can block the turnover of poly-ubiquitinated proteins through the ubiquitin-proteasome system [4, 5]. However, toxicities associated with global proteasomal inhibition and resistance to bortezomib are major concerns in MM, prompting the discovery of new agents and development of more effective therapies for the treatment of MM.

Berberine (BBR), a botanical alkaloid derived from several plural, has been traditionally used for bacterial diarrhea, anti-infection, and ocular trachoma infections in China. Accumulating studies indicated that BBR also exhibits anti-cancer activity in leukemia [6], melanoma [7], glioblastoma [8], hepatocellular carcinoma [9], colon cancer [10], and MM [11]. Several targets of BBR have been revealed. BBR can bind to RXRα in the ligand-binding domain directly and repressed β-catenin signaling in colon cancer cells [12]. BBR inhibited the activation of NF-ĸB signaling via modifying cysteine 179 of IĸBα kinase and repressed several NF-ĸB-regulated gene products (Bcl-xL, survivin, cyclin D1, and MMP-9) [13]. In MM, BBR triggered the hypomethylation of TP53 via inhibiting the expression level of DNMT1 and DNMT3B in U266 cell [14]. Our previous study reported that BBR downregulates miR-21 expression through IL6/STAT3 in MM cell lines, which resulted in the inhibition of IL-6 secretion [15]. In the ubiquitin-proteasome system, BBR plays an important role in modulating protein degradation. The Skp, Cullin, F-box containing complex-β-Transducin Repeat Containing Protein (SCF-β-TrCP) complex was recruited by BBR to promote Cyclin D1 degradation in the ubiquitin-proteasome-dependent way [16]. Fbxo32, one of the SCF ubiquitin ligase complex, is an anti-hypertrophic E3 ligase which could be upregulated by BBR to improve hypertrophic and cardiac performance in cardiac-deficient Pak1 mice [17]. BBR activated an E3 ubiquitin ligase Cbl to degrade EGFR protein, which led to cell proliferation inhibition in mouse and human colon cancer cells [18]. However, the direct targets of BBR in the ubiquitin-proteasome system remain elusive in MM.

Surface plasmon resonance (SPR) analysis is a novel bioanalytical tool to analyze the interaction between proteins, DNA, enzymes, and other biomolecules [19]. By combining SPR analysis and molecular docking, heparanase had been identified as a target of aspirin for tumor metastasis, angiogenesis, and growth in cancer [20]. Liquid chromatography-tandem mass spectrometry (LC-MS/MS) has emerged as a novel protein analytical technology applicable to protein identification. SPR-LC-MS/MS-based approach has shown great potential in target screening and identification. Ubiquitin-like with PHD and RING Finger domains 1 (UHRF1), a potential target of BBR, is highly expressed in various cancer cells, and its overexpression has been associated with tumor-promoting effects. As an epigenetic reader, UHRF1 can induce epigenetic silencing of several tumor suppressors (TSGs), including p16^INK4A^, p53, p73, and p21 [21]. As an E3 ubiquitin ligase, UHRF1 is required for tumor cell proliferation and it has been reported that UHRF1 promotes PML and P53 ubiquitination and degradation [22, 23]. A previous study revealed that high levels of CD47 connected the activation of NF-ĸB signaling pathway and overexpression of UHRF1 in glioblastoma cells [24]. Aberrant activation of NF-ĸB and CD47 has been shown to contribute to the malignant progression of MM [25, 26]. Therefore, we postulated that BBR exhibited anti-MM activity via targeting UHRF1.

This study aims to explore the target and related mechanisms involved in the anti-MM activity of BBR. SPR analysis combined with LC-MS/MS demonstrated that UHRF1, an E3 ubiquitin ligase, is a candidate target of BBR. BBR can bind UHRF1 in the tandem tudor domain (TTD) and plant homeodomain (PHD). Moreover, BBR induced UHRF1 protein degradation via the ubiquitin-proteasome system. These findings uncovered the target and molecular mechanisms involved in the anti-MM activity of BBR.

## Results

### BBR-induced inhibition of cell growth in vitro translated to anti-MM activity in vivo

**Fig. 1:**
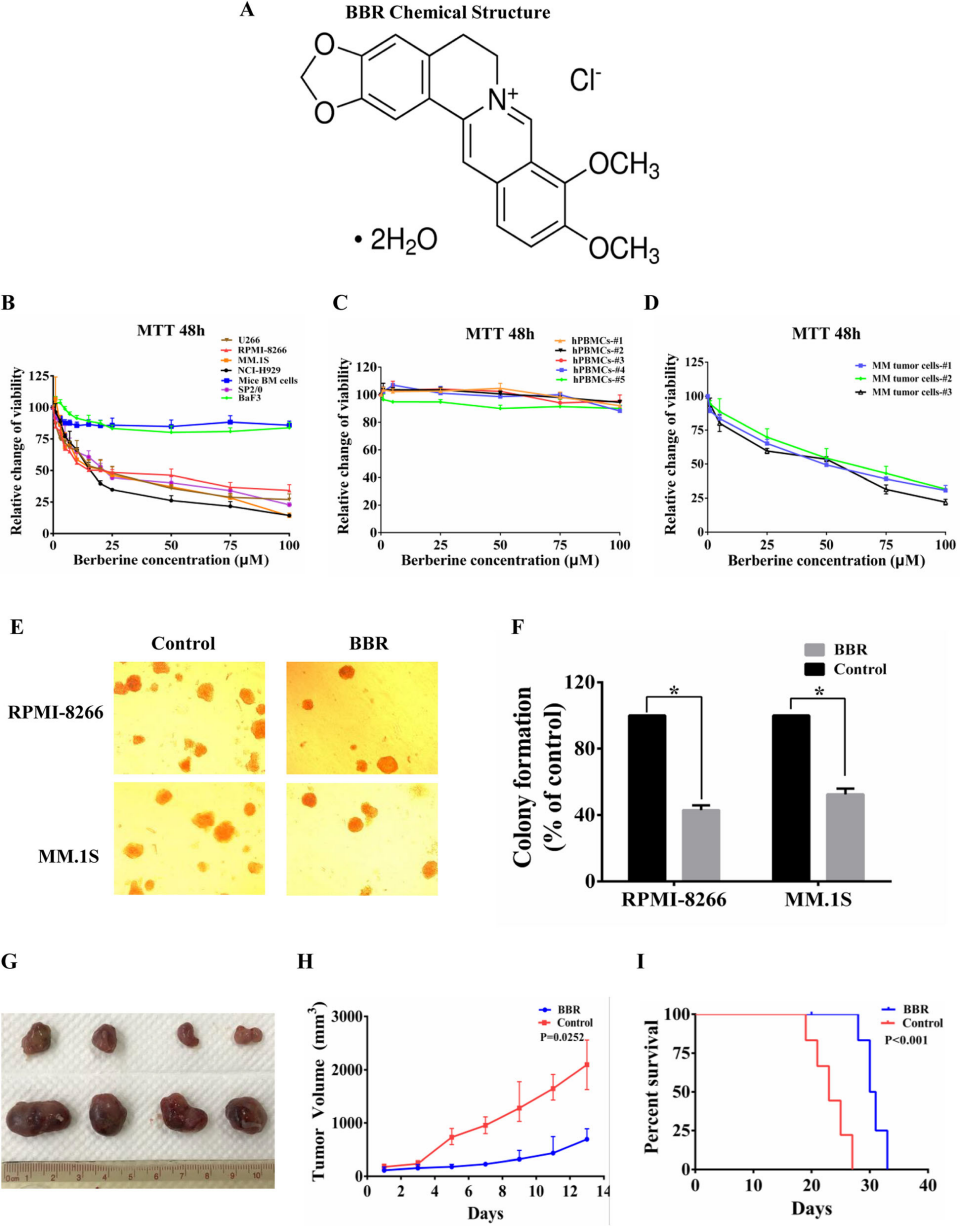
BBR-induced inhibition of cell growth in vitro translated to anti-MM activity in vivo. **a** Chemical structure of BBR. Chemical name: 1,3-benzodioxolo[5,6-a] benzo[g] quinolizinium, 5,6-dihydro-9,10-dimethoxy-, hydrochloride (1:1). Molecular formula: C20H18ClNO4. Molecular weight: 371.81. CAS number: 633-65-8. **b** BBR induced cytotoxicity in MM cell lines. Mice BM, BaF3, SP2/0, and MM cell lines were treated with BBR (0–100 μM), and viability was determined at 48 h using MTT assay. The IC50 of BBR in MM cells ranged between 15 and 25 μM at 48 h. The data were presented as the mean ± SD obtained from three independent experiments. **c** BBR activity on normal human peripheral blood mononuclear cells (hPBMCs). Normal hPBMCs were separated using Ficoll-paque density sedimentation and treated with BBR (0–100 μM) for 48 h. Cell viability was determined at 48 h using MTT assay. The data were presented as the mean ± SD obtained from three independent experiments. **d** BBR induced cytotoxicity in primary tumor cells from MM patients. Purified patient MM cells were cultured with BBR (0–100 μM), and cell viability was determined at 48 h using MTT assay. The data were presented as the mean ± SD obtained from three independent experiments. **e**, **f** BBR inhibited the colony formation ability of RPMI-8266 and MM.1S cell lines. Histogram and statistics indicated the relative number of colonies per 1000 plated cells. The data were presented as the mean ± SD obtained from three independent experiments. Significance was determined by Student’s *t* test, *p < 0.05 versus control. **g**–**i** The anti-MM activity of BBR in vivo. 2 **×** 10^7^ RPMI-8266 cells were subcutaneously injected into sub-lethally irradiated (3 Gy) BALB/c mice. Tumor-bearing mice were randomly assigned into 2 cohorts receiving either vehicle or BBR (50 mg/kg) every other day for 2 consecutive weeks. BBR treatment resulted in tumor growth inhibition. The average and SD of tumor volume (mm^3^) are shown from mice (*n* = 7/group) versus the time when the tumor was measured (*p* = 0.0252). Prolonged survival was observed in BBR treatment groups (*p* < 0.01)

BBR is an isoquinoline type of botanical alkaloid present in many traditional Chinese medicines as the main active compound (Fig. 1a). To assess the potential anti-MM activity of BBR, we investigated its in vitro effects in bone marrow (BM) cells from C57BL/6 J mice, BaF3, SP2/0, and several human MM cell lines at 48 h. Notably, the normal mice BM and BaF3 cells were not sensitive to BBR treatment. Mice myeloma cell SP2/0 and human MM cells were sensitive to treatment with BBR (Fig. 1b). In human MM cell lines, the half maximal inhibitory concentration (IC50) of BBR at 48 h ranged between 15 and 25 μM. Moreover, BBR inhibited cell growth in freshly isolated tumor cells from MM patients. BBR at the IC50 for MM cells did not affect the viability of normal human peripheral blood mononuclear cells (hPBMCs) (Fig. 1c, d). These data suggest that normal cells are completely refractory to BBR. We also investigated the effect of BBR on the colony formation ability of MM cells, and BBR treatment significantly reduced the number of RPMI-8266 and MM.1S cell colonies versus control (Fig. 1e, f). Next, the effect of BBR and bortezomib on the proteasome activity and ubiquitinated protein level was compared in MM cells. This analysis showed that bortezomib induced a marked increase in ubiquitylated proteins than BBR, whereas a modest increase in polyubiquitylation was observed in BBR-treated cells (Additional file 1, Figure S1). This may be attributed to the narrow effects of BBR on proteasome activity, while bortezomib targeted proteasome. These data suggest that the targets and molecular mechanism of the anti-MM activity of BBR may be different from bortezomib.

The in vitro BBR-induced cell growth inhibition to MM cells translated into effective in vivo anti-MM activity in RPMI-8266 xenograft model. BBR treatment significantly suppressed the growth of MM tumors, with the maximum inhibition of tumor growth (66.71%) noted at day 14 in the cohorts treated with 50 mg/kg BBR (Fig. 1g, h). Treatment with BBR was also associated with improved survival. The first deaths in the control and BBR-treated mice were noted at days 19 and 28, respectively (Fig. 1i). These data indicate that BBR may be a promising drug for the treatment of MM.

### Screening the potential targets of BBR via using SPR-LC-MS/MS approach

**Fig. 2:**
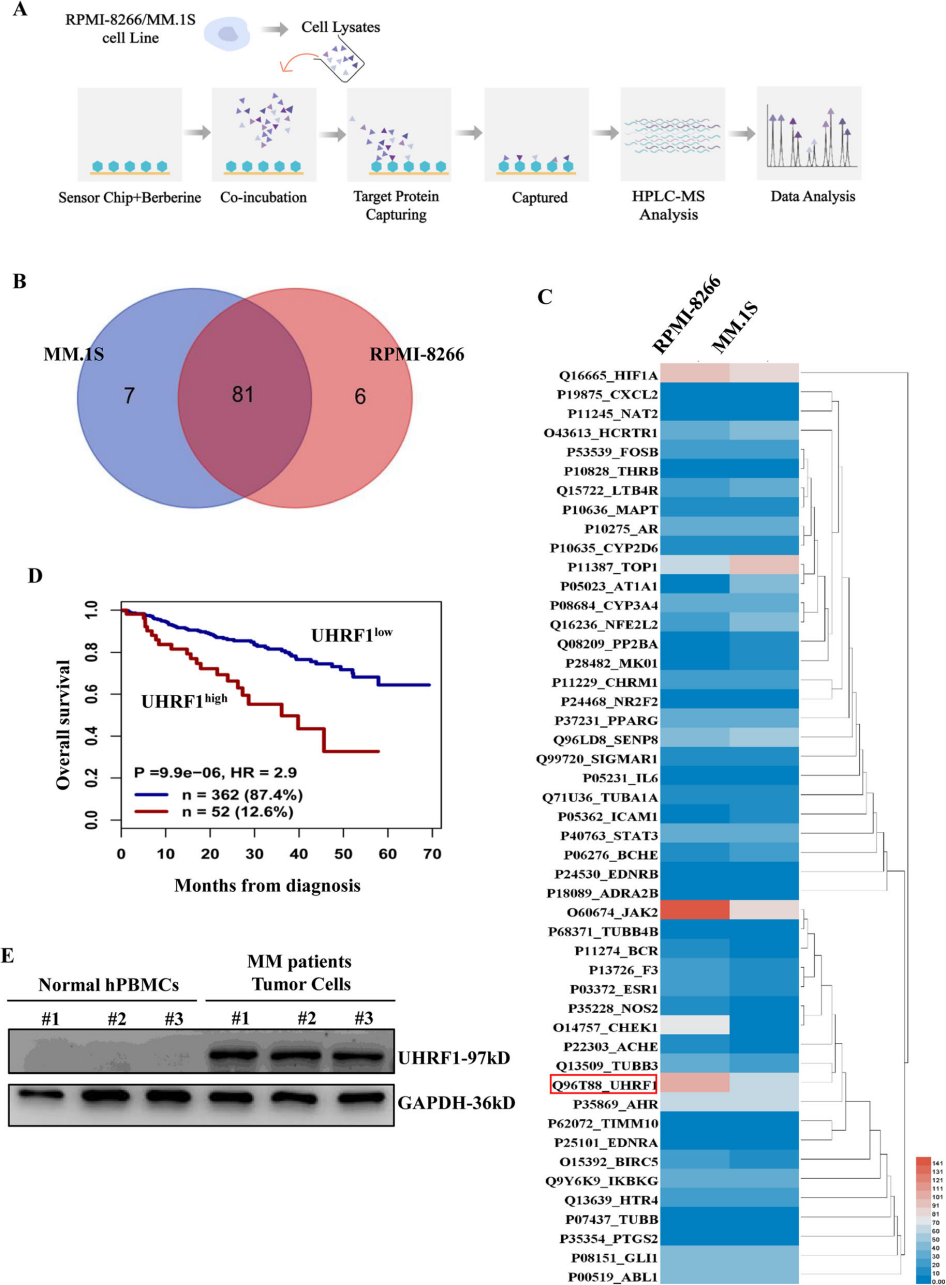
Screening the potential targets of BBR via SPR-LC-MS/MS approach. **a** The schematic generation of SPR-LC-MS/MS approach. BBR is immobilized on a sensor chip and incubated with the cell lysates from RPMI-8266 and MM.1S cells, followed by SPR analysis. The potential targets of BBR from BBR-protein mixtures were screened and ranked by mass spectrometry analysis. **b** Venn diagram showing 81 proteins commonly identified in MM cells by SPR-LC-MS/MS approach. A total of 88 and 87 proteins were identified from MM.1S and RPMI-8266 cells in the mass spectrometry analysis, respectively. **c** Forty-eight potential targets of BBR are shown in the relative quantity heatmap by using a score value cutoff > 700. **d** Prognostic value of UHRF1 mRNA levels in terms of overall survival in newly diagnosed patients from the UAMS TT2 (*n* = 256) and UAMS TT3 (*n* = 158) cohorts. Maxstat analysis was used to calculate the optimal separation of patients based on a cutoff value. **e** UHRF1 expression in MM primary tumor cells and normal hPBMCs. Normal hPBMCs and MM primary tumor cells were separated using Ficoll-paque density sedimentation. Cells lysates were harvested and subjected to western blotting with anti-UHRF1 and anti-GAPDH antibodies

To investigate the direct targets and molecular mechanism of BBR, SPR-LC-MS/MS approach was performed to screen and rank the direct targets required for the anti-MM activity of BBR (Fig. 2a). BBR is immobilized on a sensor chip and incubated with the cell lysates from RPMI-8266 and MM.1S cells, followed by SPR analysis. The potential targets of BBR from BBR-protein mixtures were ranked by mass spectrometry analysis. A total of 88 and 87 proteins in MM.1S and RPMI-8266 cells were identified through SPR-LC-MS/MS approach, respectively. Eighty-one proteins were commonly identified, indicating that BBR may have conserved targets in MM cells (Fig. 2b). The score and PSMs (Peptide-Spectrum Matches) of captured proteins are demonstrated in Additional file 2, Table S1; Additional file 3, Table S2; and Additional file 4, Table S3. Using a score value cutoff > 700, we screened 48 potential targets of BBR. The relative quantity heatmap of 48 captured target proteins is shown in Fig. 2c. JAK2, UHRF1, and HIF1A are the top 3 proteins (score value cutoff > 700, PSM value cutoff > 60), according to mass spectrum analysis and heatmaps. UHRF1, the only target related to the ubiquitin-proteasome system pathway in mass spectrometry analysis, was selected for further study.

Next, we investigated the relation between *UHRF1* expression and disease outcome using 2 cohorts of newly diagnosed MM patients (GSE4581). By using the Maxstat R package, MM patients were divided into UHRF1 high (*n* = 51) and low (362) expressers. In the UAMS TT2 and TT3 cohorts, high expression of UHRF1 had a bad prognosis (Fig. 2d). We also compared the expression levels of UHRF1 protein in MM primary tumor cells and MM cell lines with normal hPBMCs. It showed that UHRF1 was barely detected in normal hPBMCs. The expression level of UHRF1 was higher in the MM primary tumor and MM cells compared to normal hPBMCs (Fig. 2e; Additional file 5, Figure S2). Together, these data show that *UHRF1* is upregulated in MM and correlates with a poor prognosis. This indicates that UHRF1 may play an oncogenic role in MM.

### BBR directly binds to UHRF1 in the TTD-PHD domain

**Fig. 3:**
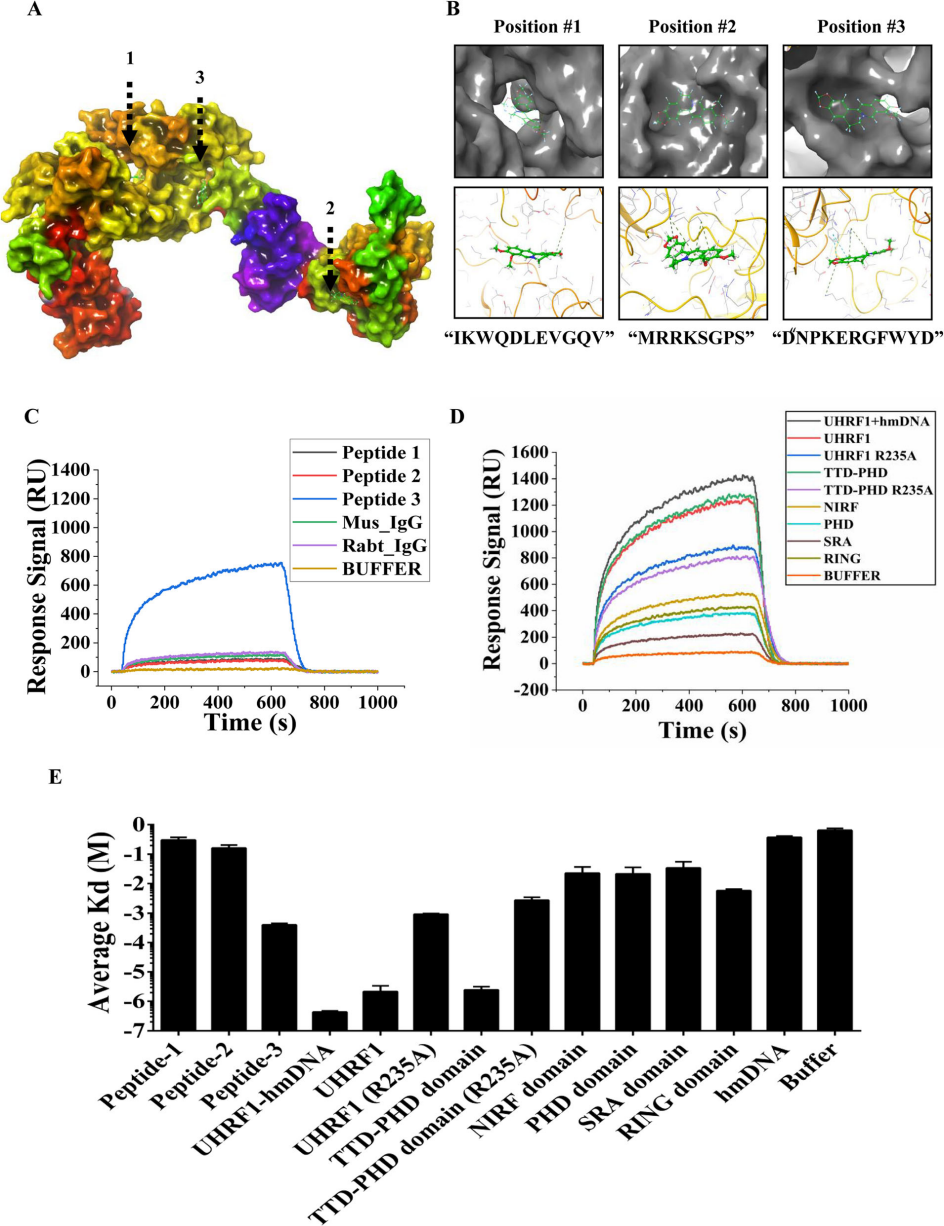
BBR directly binds to UHRF1 in the TTD-PHD domain. **a** Structural overview of a UHRF1-BBR complex model predicted based on information from the competitive molecular docking experiment. There are three active sites in the UHRF1-BBR complex model. **b** Zoom-in view of the predicted active-site peptides (“IKWQDLEVGQV,” “MRRKSGPS,” and “PDNPKERGFWYD”). Key interface residues (D216, K297, and R235) in UHRF1 are shown. **c** Binding response curves of interactions between BBR and peptide 1/2/3. Peptide 3-BBR interaction was validated by surface plasmon resonance analysis. **d** Binding response curves of interactions between BBR and different domains of UHRF1 or hm-DNA. Proteins were purified from *E. coli* lysates overexpressing different domains of UHRF1. The BBR-UHRF1, BBR-TTD PHD domain, and BBR-hmDNA+UHRF1 interaction were validated by surface plasmon resonance analysis. **e** The average Kd values were measured in the surface plasmon resonance analysis. The data were presented as the mean ± SD obtained from three independent experiments

To study the interaction of BBR and UHRF1, we used the Maestro software to model the structure of UHRF1. UHRF1 domains were obtained from the Protein Data Bank (http://www.rcsb.org/pdb/home/home.do). The amino acid gaps were automatically filled using the homology modeling program. There were three active sites in the BBR-UHRF1 complex model (Fig. 3a). The molecular docking model suggested that the binding site of BBR on UHRF1 consists of the following residues: peptide 1 “IKWQDLEVGQV,” peptide 2 “MRRKSGPS,” and peptide 3 “PDNPKERGFWYD.” The key interface residues in the above peptides were Aspartic acid 216 (D216), Lysine 297 (K297), and Arginine 235 (R235), respectively (Fig. 3b).

To further determine whether BBR can directly bind to these three peptides, we obtained synthetic peptides and performed SPR analysis. BBR was strongly bound to peptide 3, but not to peptide 1/2 (Fig. 3c). The average Kd of BBR-peptide 3 was 3.68E−04, and the intensity of interaction was middle (Additional file 6, Table S4). Based on the evidence above, BBR may directly interact with peptide 3, which was located in the TTD-PHD domain. Molecular docking had shown that BBR can bind to the TTD-PHD domain (Additional file 7, Figure S3). To further study the UHRF1-BBR interaction, proteins were purified from *Escherichia coli* lysates overexpressing different domains of UHRF1 (Additional file 8, Figure S4) and SPR analysis was performed. It showed that BBR can interact with UHRF1-TTD-PHD domain and UHRF1 protein (Fig. 3d). The average Kd of BBR-UHRF1 and the BBR-TTD-PHD domain was 1.30E−06 and 2.96E−06, respectively (Fig. 3e, Additional file 6, Table S4). The intensity of BBR-UHRF1 and BBR-TTD-PHD domain interaction was strong. However, the point mutant R235A of UHRF1 or TTD-PHD did not exhibit a strong intensity of interaction with BBR. Hemi-methylated DNA has been shown to open the UHRF1 closed conformation to allow accessibility of TTD to its target epigenetic mark H3K9me2/3 [27], and we performed SPR analysis after pre-incubated UHRF1 with hemi-methylated DNA at 4 °C for 10 min. The incubation of UHRF1 with hmDNA promotes the BBR-UHRF1 interaction, and the average Kd of BBR-UHRF1-hmDNA was 4.60E−07 (Fig. 3e; Additional file 6, Table S4). It meant that the Kd of BBR for UHRF1 is the true value. These data suggest that BBR interacted with UHRF1 through its TTD-PHD domain.

### BBR promotes UHRF1 protein degradation and reactivates several TSGs

**Fig. 4:**
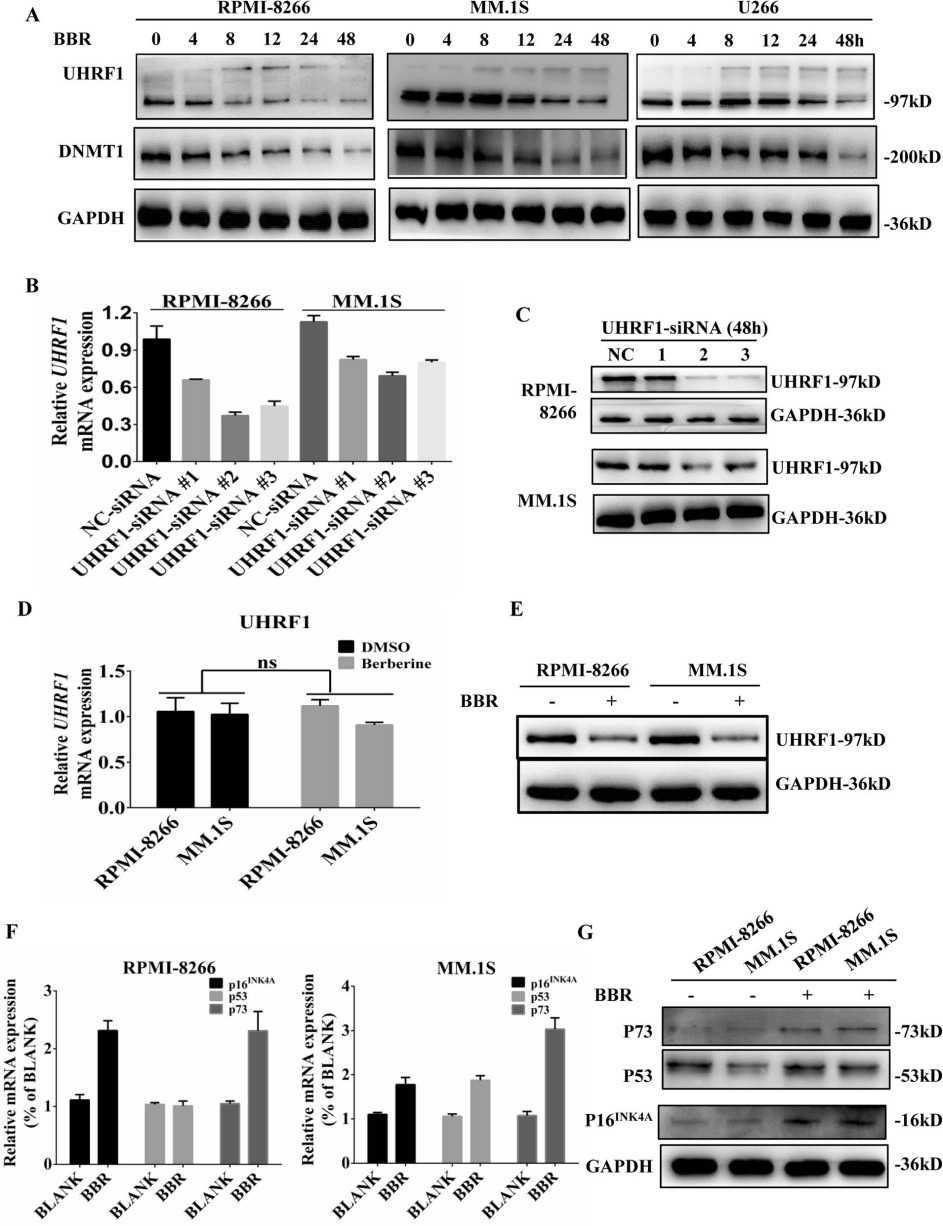
BBR promotes UHRF1 protein degradation and reactivates several TSGs. **a** BBR reduced the expression of UHRF1 and DNMT1 proteins. UHRF1 in RPMI-8266, MM.1S, and U266 cells were treated with 25 μM BBR for 0, 4, 8, 12, 24, and 48 h. Cell lysates were harvested and subjected to western blotting with anti-UHRF1, anti-DNMT1, and anti-GAPDH antibodies. **b**, **c** Screening the effective UHRF1-siRNA in MM cells. UHRF1-siRNA #2 was confirmed as the effective UHRF1-siRNA through qRT-PCR and western blotting after transfection with 100 nM UHRF1-siRNAs for 48 h. The data were presented as the mean ± SD obtained from three independent experiments. **d**, **e** The effect of BBR on the expression of UHRF1 at 48 h. The mRNA and protein expression of UHRF1 in MM cells was determined through qRT-PCR and western blotting after treatment with or without 25 μM BBR for 48 h. The data were presented as the mean ± SD obtained from three independent experiments. **f**, **g** The effect of BBR on the expression of p16^INK4A^, p53, and p73. The mRNA and protein expression of p16^INK4A^, p53, and p73 in MM cells were determined through qRT-PCR and western blotting after treatment with or without 25 μM BBR for 48 h. The data were presented as the mean ± SD obtained from three independent experiments

To test whether BBR could downregulate UHRF1 in MM cells, we treated MM cells with BBR for indicated time, and then, cell lysates were harvested and subjected to western blotting analysis. As shown in Fig. 4a, BBR inhibited the expression of UHRF1 protein in MM cell lines in a time-dependent way. BBR treatment also resulted in the occurrence of smear-like band (a hallmark of post-translational modification including ubiquitination) between 130 and 170 kDa in the tested cell lines about 8 h. DNA methyltransferase 1 (DNMT1), an important substrate of UHRF1, was also inhibited by BBR in MM cell lines. Next, we screened effective UHRF1-siRNA #2 on UHRF1 mRNA and protein from three siRNAs as a positive control, which was used to compare the effect of BBR on UHRF1 mRNA and protein level (Fig. 4b, c). Treatment with 25 μM BBR for 48 h inhibited UHRF1 protein expression but not on *UHRF1* mRNA (Fig. 4d, e). Collectively, these data demonstrate that BBR induces the downregulation of UHRF1 proteins via a post-transcriptional mechanism. Arginine 235 has been shown as the key interface residue of BBR-UHRF1 interaction. Here, to evaluate this mutant response to BBR treatment, we transfected UHRF1-R235A and UHRF1 plasmids into NIH-3 T3 cells. R235A in UHRF1 abolished the effect of BBR on UHRF1 protein, suggesting that the key interface residue R235 in the TTD-PHD domain is responsible for BBR-induced degradation of UHRF1 (Additional file 9, Figure S5). MM patients with high levels of tumor suppressor genes (TSGs) may have a relatively indolent form of the disease, with good prognostic features and long overall survival [28]. Several TSGs, including p16^INK4A^, p53, and p73, could be silenced by UHRF1. BBR can re-activate p16^INK4A^, p53, and p73 in MM.1S, and p16^INK4A^ and p73 in RPMI-8266 cells (Fig. 4f, g). This different mRNA and protein change on P53 may be due to RPMI-8266 carrying the p53 mutant.

### BBR induces UHRF1 degradation through ubiquitin-proteasome system pathway

**Fig. 5:**
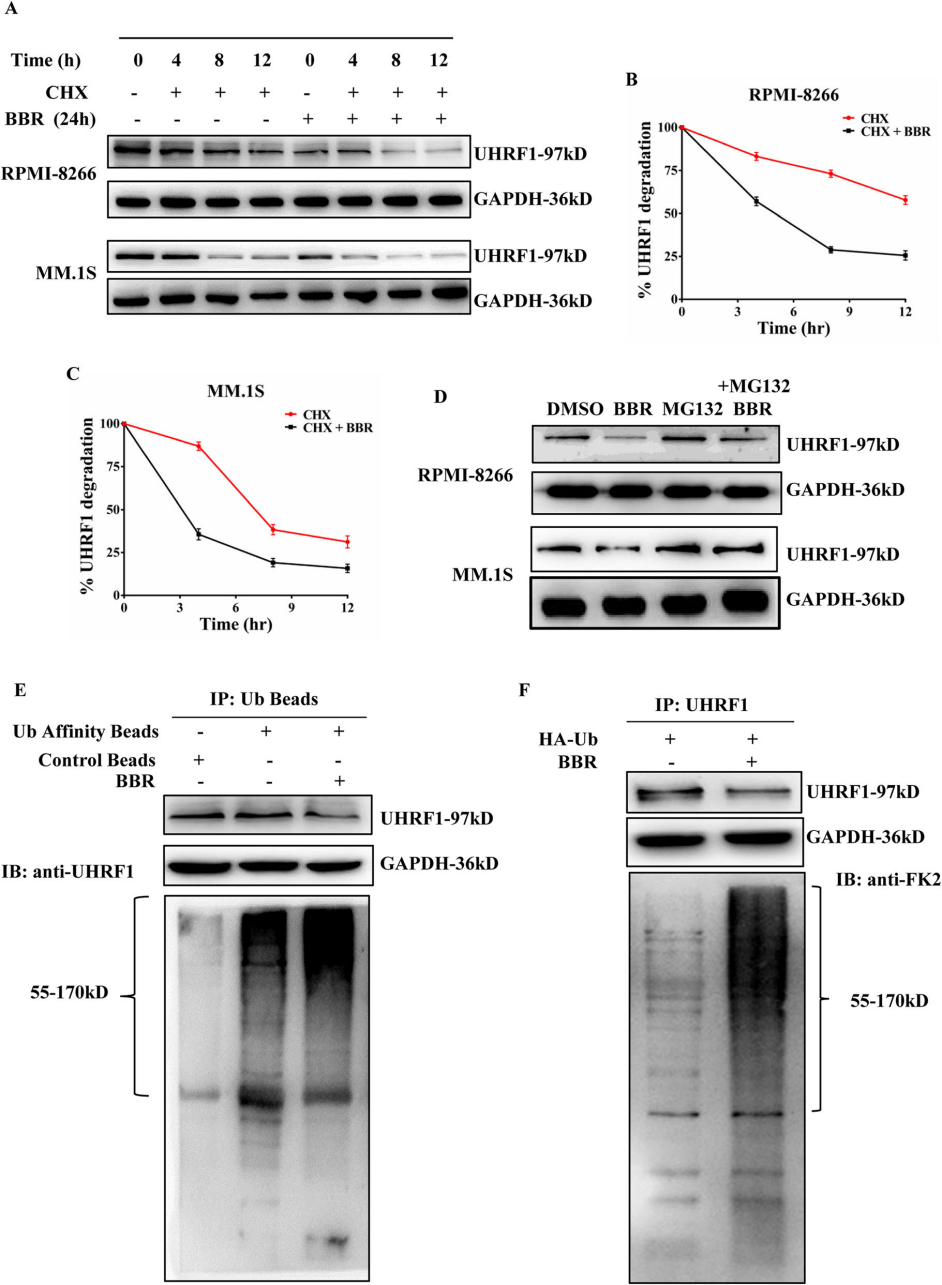
BBR induces UHRF1 degradation via the ubiquitin-proteasome system pathway. **a** BBR affected the stability of UHRF1 in MM cells. RPMI-8266 and MM.1S cells were treated with DMSO alone, BBR (25 μM) alone for 24 h, or pretreated with DMSO or BBR for 12, 16, and 20 h, followed by addition of CHX (50 μg/mL) for additional 4, 8, and 12 h. Cell lysates were harvested and subjected to western blotting with anti-UHRF1 and anti-GAPDH antibodies. **b**, **c** Densitometry was utilized to quantify UHRF1 protein levels after normalization with GAPDH control to obtain percent UHRF1 degradation in RPMI-8266 and MM.1S cells. The data were presented as the mean ± SD obtained from three independent experiments. **d** MG132 abolished the effect of BBR on UHRF1 degradation. RPMI-8266 and MM.1S cells were treated with DMSO alone, BBR (25 μM) alone for 24 h, or pretreated with DMSO or BBR for 20 h, followed by addition of MG132 for additional 4 h. Cell lysates were harvested and subjected to western blotting with anti-UHRF1 and anti-GAPDH antibodies. **e** RPMI-8266 cells were subsequently treated with BBR (25 μM for 24 h) prior to harvesting. The proteins modified by ubiquitination were purified from cell extracts using anti-UB beads and subjected to western blotting with anti-UHRF1 and anti-GAPDH antibodies. **f** RPMI-8266 cells were transiently transfected with HA-Ub constructs, and endogenous UHRF1 proteins were immunoprecipitated from BBR-treated or BBR-untreated RPMI-8266 cells (25 μM for 24 h). Immunoprecipitates were harvested and subjected to western blotting with anti-UHRF1, anti-FK2, and anti-GAPDH antibodies

Next, we investigated the effect of BBR on the stability of UHRF1 proteins, pretreated MM.1S and RPMI-8266 cells with BBR for the indicated time, followed by cycloheximide (CHX) addition. BBR treatment resulted in the increased degradation of UHRF1 versus control cycloheximide (CHX)-alone-treated MM cells (Fig. 5a). The half-life of UHRF1 is about 14 h in RPMI-8266 cells and 8 h in MM.1S cells (Fig. 5b, c). Considering that BBR affected the stability of UHRF1, we subsequently examined the catabolic properties of this E3 ligase using inhibitors against lysosome (Chloroquine), autophagy (3-MA), and proteasome pathways (MG132). MG132, unlike the other inhibitors, caused an obvious increase of UHRF1 (Fig. 5d; Additional file 10, Figure S6). These findings indicate that BBR degraded UHRF1 mainly in the context of the proteasome system.

To examine the ubiquitination of UHRF1 in MM cells, an analysis of the affinity enrichment of ubiquitin-modified proteins was performed on lysates from BBR-treated or BBR-untreated RPMI-8266 cells. A marked increase in UHRF1 ubiquitination was detected in the BBR-treated cells (Fig. 5e). Furthermore, RPMI-8266 cells were transiently transfected with HA-Ub constructs, and endogenous UHRF1 proteins were immunoprecipitated with/without BBR treatment in RPMI-8266 cells. As expected, we found that the levels of UHRF1 ubiquitination were higher in BBR-treated RPMI-8266 cells (Fig. 5f). Collectively, these results suggest that BBR promotes the degradation of UHRF1 through the ubiquitin-proteasome system.

### In vitro effects of UHRF1 on the proliferation of MM cells

**Fig. 6:**
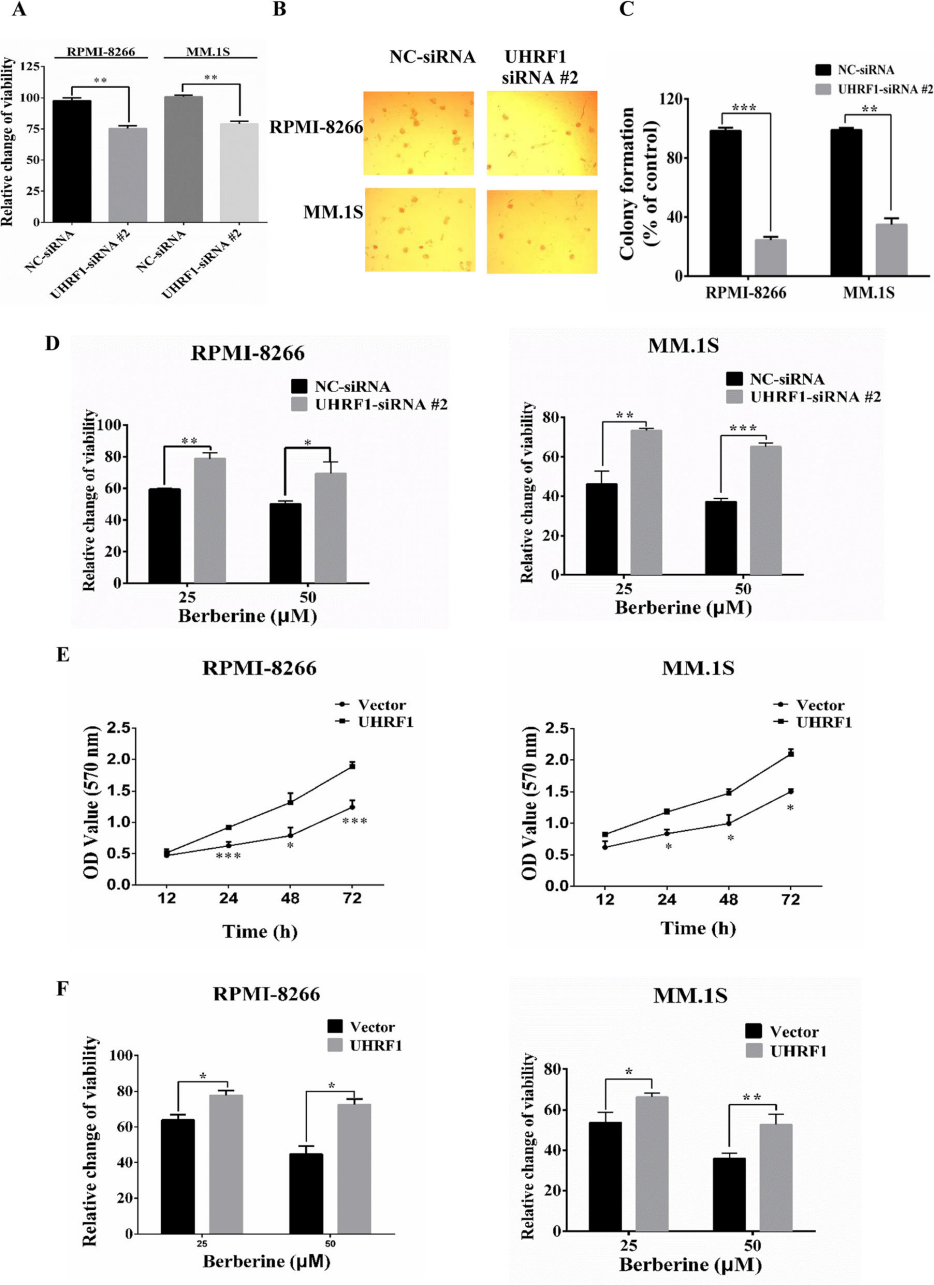
The in vitro effects of UHRF1 on the proliferation of MM cells. **a** RPMI-8266 and MM.1S cells were transfected with either NC or UHRF1 siRNA #2100 nM for 48 h, and cell viability was determined using MTT assay. The data were presented as the mean ± SD obtained from three independent experiments. Significance was determined by Student’s *t* test, **p < 0.01 versus NC-siRNA transfection groups. **b**, **c** Targeting of UHRF1 with UHRF1-siRNA #2 transfection inhibited the colony formation ability of RPMI-8266 cells. Histogram and statistics indicating the relative number of colonies per 1000 plated cells. The data were presented as the mean ± SD obtained from three independent experiments. Significance was determined by Student’s *t* test, **p < 0.01, ***p < 0.001 versus NC-siRNA transfection groups. **d** RPMI-8266 and MM.1S cells were transfected with NC-siRNA or UHRF1-siRNA #2100 nM for 24 h, cells were subsequently treated with DMSO or BBR (25, 50 μM) for 24 h, and cell viability was determined using MTT assay. Percent cell viability was normalized (as 100%) for NC- or UHRF1-siRNA #2 controls, respectively. The data were presented as the mean ± SD obtained from three independent experiments. Significance was determined by Student’s *t* test, *p < 0.05, **p < 0.01, ***p < 0.001 versus NC-siRNA transfection groups. **e** Overexpression of UHRF1 promoted the cell proliferation of RPMI-8266 and MM.1S. RPMI-8266 and MM.1S cells were transfected with Lentiviral Flag-UHRF1 or vector carrying a puromycin selection marker. After puromycin selection for 2 weeks, cell viability was determined at 12 h, 24 h, 48 h, and 72 h using MTT assay. The data were presented as the mean ± SD obtained from three independent experiments. Significance was determined by Student’s *t* test, *p < 0.05, ***p < 0.001 versus vector. **f** Overexpression of UHRF1 renders RPMI-8266 and MM.1S cells more resistant to BBR. RPMI-8266 and MM.1S cells were transfected with Lentiviral Flag-UHRF1 or vector carrying a puromycin selection marker. After puromycin selection for 2 weeks, cell viability was determined at 48 h using MTT assay. The data were presented as the mean ± SD obtained from three independent experiments. Significance was determined by Student’s *t* test, *p < 0.05, **p < 0.01 versus vector

To investigate whether UHRF1 is involved in the increased growth of MM cells, UHRF1-siRNA #2 were transfected into RPMI-8266 and MM.1S cells; MTT assay was used to assess the cell viability. It showed that transfection with 100 nM UHRF1-siRNA #2 for 48 h decreased the cell abilities versus NC-siRNA in RPMI-8266 and MM.1S cells (Fig. 6a). Transfection with UHRF1-siRNA #2 also reduced the colony formation ability of RPMI-8266 and MM.1S cells significantly versus that observed in cells transfected with NC-siRNA (Fig. 6b, c). To test whether the levels of UHRF1 protein influence the anti-proliferative effect of BBR, RPMI-8266 and MM.1S cells were transfected with UHRF1-siRNA #2 or NC-siRNA 24 h, followed by treatment with BBR for 24 h, and then analyzed for viability. Knockdown UHRF1 expression with siRNA #2 attenuated BBR-induced cytotoxicity in MM cells (Fig. 6d). On the other hand, the overexpression of UHRF1 in RPMI-8266 and MM.1S cells could enhance cell growth (Fig. 6e) and the overexpression of UHRF1 in MM cells were confirmed by western blotting (Additional file 11, Figure S7). We subsequently tested whether the overexpression of UHRF1 would render MM cells more resistant to BBR. As shown in Fig. 6f, RPMI-8266 and MM.1S cell line with stable overexpression of UHRF1 was more resistant to BBR than the parental cells. Together, these data indicate that BBR induced cytotoxicity in MM cells via targeting UHRF1.

## Discussion

The aim of this study was to uncover the direct targets and molecular mechanism of berberine (BBR) in multiple myeloma (MM). Approximately 90% of MM patients aged > 50 years are not eligible for high-dose therapy [1]. The novel anti-MM agents (including proteasome inhibitors, histone deacetylase inhibitors, immunomodulators, and monoclonal antibodies) have been approved for the treatment of MM [29]. However, long-term use was often associated with the development of resistance and occurrence of relapse. The ancient herbal medicines may lead to the discovery of novel strategies for the treatment of MM, taking into account the age of MM patients and their tolerability of chemotherapy. BBR has been used historically in anti-inflammatory therapy in China, and the progression of MM is accompanied with higher levels of inflammatory cytokines (e.g., IL-6 and IL-18) [30]. We demonstrated that treatment of MM cells with BBR resulted in MM cytotoxicity in vitro associated with inhibition of MM tumor growth and prolonged survival in vivo. However, it currently lacks screening and identifying the direct targets required for the anti-MM activity of BBR.

SPR-LC-MS/MS-based approach was performed to screen and rank the potential targets of BBR in MM cells, and 81 targets have been identified in mass spectrum analysis. Of these molecules, JAK2, UHRF1, and HIF1A are the top 3 proteins which are considered as the BBR-binding proteins. Recently, BBR has been reported to inhibit the phosphorylation of JAK/STAT3 signaling without altering the total proteins of JAK2 and STAT3 to protect rat heart from ischemia/reperfusion injury [31], suggesting that JAK2 may not be the direct target of BBR. Fu et al. also showed that BBR treatment can significantly inhibit the expression of HIF-1α at protein and mRNA levels [32]. It indicated that BBR may inhibit the transcription of HIF1A through unknown regulatory mechanisms. The abnormality of ubiquitin-proteasome system is linked to the pathogenesis of various human diseases, especially MM [33]. Bortezomib therapy in MM is associated with broader substrate spectrum and drug resistance [34]. Accumulated studies have concerned more about another functional molecular in the ubiquitin-proteasome system, rather than proteasome itself. Deubiquitylating enzymes, E1-conjugating enzymes, or E3 ubiquitin ligases have emerged as the potential therapeutic targets for cancer treatment [33, 35, 36]. Ubiquitin-like with PHD and RING Finger domains 1 (UHRF1) is the only potential target related to the ubiquitin-proteasome system in mass spectrometry identification. UHRF1 plays an important role in DNA CpG methylation, cell proliferation, ubiquitin-proteasome system, and gene expression [21]. The interaction of BBR-UHRF1 was confirmed by molecular docking and SPR analysis. It has been shown that UHRF1 conformation could be regulated by hm-DNA, and this step promotes histone H2K9me3 recognition by UHRF1 at the TTD domain [27]. The pre-incubation of UHRF1 with hmDNA can enhance the BBR-UHRF1 interaction. Thus, we believe that BBR-UHRF1 binding is real and UHRF1 is the direct target required for the anti-MM activity of BBR.

In vitro and in vivo studies have shown that a drug-induced inhibition of UHRF1 activity or expression leads to the reactivation of tumor suppressor genes, enabling cancer cells to undergo apoptosis and cell cycle arrest [37]. MM is considered as the highly heterogeneous cancer at the genetic levels. Genetic abnormalities and epigenetic aberrations played an important role in the progression of MM and drug resistance. The change of DNA methylation, histone modifications of genes, and tumor suppressor genes also can be involved in the MM resistance mechanism [28]. BBR treatment can inhibit UHRF1 protein levels in MM cells but no alteration at mRNA level, which indicated that the regulation on UHRF1 is at post-transcriptional modification. DNMT1, a known substrate of UHRF1 [38], was also degraded by BBR treatment. And several tumor suppressor genes (p16^INK4A^ and p73) regulated by UHRF1 could be re-activated by BBR in MM cells. Of note, UHRF1 protein expression is much higher in primary MM tumor and MM cells compared to normal hPBMCs, and with an unfavorable prognosis in MM. Knockdown of UHRF1 expression in cancer cells significantly suppressed cell growth, indicating that UHRF1 is essential for the progression of cancer [39]. Interestingly, normal cells (BaF 3 and normal hPBMCs) expressing low UHRF1 are insensitive to BBR in contrast to MM cells which supports that UHRF1 may be the target of BBR. Targeting UHRF1 expression in MM cells caused the inhibition of cell growth and colony formation, and the alteration of UHRF1 protein levels is related to the cytotoxicity induced by BBR. Collectively, the results of these studies have led to the proposal that UHRF1 is a tumor biomarker and therapeutic target for MM.

Several natural compounds (e.g, thymoquinone, anisomycin, and luteolin) have been reported to affect the expression of UHRF1 on mRNA and protein [40–42]. Nevertheless, the mechanism of UHRF1 downregulation induced by these natural compounds remains to be further deciphered. In this study, we demonstrated that BBR exerts its anti-MM effects through the ubiquitin-proteasome system, ultimately leading to the degradation of UHRF1 directly, which is different from the inhibitory effect of bortezomib on proteasome activity. However, the explicit biological role mechanism of UHRF1 in MM remains to be further studied. Further investigation on the detailed biological function of UHRF1 and the anti-tumor effects of berberine-based treatments in preclinical models of human MM should be carried out.

## Conclusion

In summary, this study has illustrated UHRF1 as a target of BBR. BBR may directly bind to the TTD-PHD domain to induce its degradation via the ubiquitin-proteasome system, thereby upregulating several tumor suppressor genes and impeding cell growth both in vitro and in vivo. Our findings provide insight into the molecular mechanisms and target of BBR, which may further open its therapeutic applications in MM treatment.

## Methods

### Materials

Antibodies, mouse models, chemicals, recombinant proteins, plasmids, PCR primers, and oligonucleotides used in this study are listed in Additional file 12, Table S5; Additional file 13, Table S6; Additional file 14, Table S7; and Additional file 15, Table S8.

### Cells culture

MM.1S, RPMI-8266, U266, NCI-H929, OPM2, and SP2/0 cell lines were obtained from the Institute of Shanghai Cell Biology, China. BaF3 cells were kindly presented by Professor Wenli Feng, Chongqin Medical University, China. MM cells were cultured in RPMI-1640 medium (Gibco) supplemented with 10% fetal bovine serum (FBS) and antibiotics (100 U/mL penicillin and 100 mg/mL streptomycin) at 37 °C in a 5% CO_2_ humidified atmosphere. BaF3 cells were cultured in RPMI-1640 medium (Gibco) supplemented with 10% fetal bovine serum (FBS), mouse IL-3 (1 nM), and antibiotics (100 U/mL penicillin and 100 mg/mL streptomycin) at 37 °C in a 5% CO_2_. NIH-3 T3 cell line was obtained from the Institute of Shanghai Cell Biology, China. NIH-3 T3 cells were cultured in Dulbecco’s modified Eagle’s medium (DMEM; Gibco) supplemented with 10% FBS and antibiotics (100 U/mL penicillin and 100 mg/mL streptomycin) at 37 °C in a 5% CO_2_ humidified atmosphere. Cells from C57BL/6 J mice bone marrow (femoral bone) were mechanically dissociated, and the red blood cells were removed using red blood cell lysis buffer (CWBIO, Jiangsu, China). Peripheral blood samples were obtained from healthy adult donors at the Guangdong Provincial Emergency Hospital/Guangdong Second Provincial General Hospital (Guangzhou, China) after obtaining written informed consent. The study was conducted according to the institutional guidelines and the principles of the Declaration of Helsinki. Patient MM primary tumor cells were obtained from bone marrow (BM) aspirates from the Third Affiliated Third Hospital of Southern Medical University (Guangzhou, China) after obtaining written informed consent. hPBMCs and BM mononuclear cells were separated using Ficoll-paque density sedimentation, and plasma cells were purified by positive selection with anti-CD138 magnetic activated cell separation microbeads (Miltenyi Biotec, San Diego, CA, USA).

### Transfection

For transient transfection, MM cells were transiently transfected with negative control (NC) siRNA or UHRF1 siRNAs (RiboBio Co., Ltd., Guangzhou, China). All RNA duplexes (100 nM) were transfected into MM cells using Lipofectamine™ 2000 according to the instructions provided by the manufacturer. Lentiviral infection to establish RPMI-8266/MM.1S cell lines stably expressing UHRF1 was performed according to the manufacturer’s instructions (FulenGen Co., Ltd., Guangzhou, China).

### Cell viability assay

After treatment with BBR or UHRF1 siRNA transfection for indicated time, cell viability was determined by 3-(4,5-dimethyl-thiazol-2-yl)-2,4-diphenyl-tetrazolium bromide (MTT) assays. Briefly, MM cells were seeded at a density of 1 × 10^5^ cells/mL in 96-well plates (100 μL/well). MTT stock solution (5 mg/mL) (10 μL) was added to each well, and the plate was incubated for 4 h at 37 °C. The medium was subsequently removed, and dimethyl sulfoxide (DMSO) (150 μL) was added to dissolve the blue formazan crystals produced by viable cells. Cell viability was assessed by measuring the absorbance at 570 nm on a Bio-Rad microtiter plate reader (Bio-Rad, CA, USA).

### Colony formation assay

Cells treated with BBR or UHRF1 siRNA #2 were seeded onto a 24-well plate (1 × 10^3^ cells per well) and thoroughly mixed with 0.9% methylcellulose solution in RPMI-1640 containing 20% FBS. Single cells were randomly and evenly distributed in each well. Colonies were formed during incubation for 1–2 weeks at 37 °C in a 5% CO_2_ humidified atmosphere. Light microscopy was used to observe and count the colonies containing > 50 cells.

### Proteasome activity assay and quantitative RT-PCR

The proteasome activity was measured using a 20S Proteasome Activity Assay Kit (APT280; Millipore, Billerica, MA, USA) following the manufacturer’s instructions. Total RNA was isolated and extracted using TRIzol (Invitrogen). Following reverse transcription, the mRNAs were detected using SYBR-Green real-time PCR assays. The levels of mRNA expression were normalized to those of *GAPDH*, and the fold change in mRNA levels was calculated using the 2^−ΔΔCT^ method.

### Western blotting, immunoprecipitation (IP), and co-immunoprecipitation (co-IP)

Different cells were lysed on ice in cell lysis buffer containing PMSF (Beyotime Biotechnology, Shanghai, China) for 30 min and subsequently centrifuged at 13,000 rpm for 30 min at 4 °C. For western blotting, the protein concentrations were quantified using the Bio-Rad Protein Assay Reagent (Bio-Rad, CA, USA) according to the protocol provided by the manufacturer. For IP, clarified cell lysates were incubated with 15 μL Protein G plus/Protein A-agarose and 1 μg of antibodies overnight at 4 °C. IP, co-IP, and western blotting samples were resolved using sodium dodecyl sulfate-polyacrylamide gel electrophoresis (SDS-PAGE) and transferred to polyvinylidene difluoride membranes (Merck Millipore, Darmstadt, Germany). After washing, the blots were incubated with primary antibody (Additional file 12, Table S5), followed by HRP-conjugated secondary antibody (Additional file 12, Table S5). Signals were visualized using enhanced chemiluminescence (Merck Millipore, Darmstadt, Germany) and analyzed using a UVITEC Alliance 4.7 gel imaging system (Cambridge, UK).

### Protein expression and purification

Plasmids encoding hexa-histidine-tagged recombinant human UHRF1 or UHRF1-R235A protein and its domains (i.e., NIRF, TTD-PHD, TTD-PHD-R235A, PHD, SRA, and RING) were transformed into *Escherichia coli* (BL21 (DE3)). After bacterial growth to an absorbance of 0.4–0.6 at 600 nm in Terrific Broth containing 30 mg/L kanamycin at 37 °C, induction was performed at 18 °C using 0.5 mM isopropyl-β-d-thiogalactoside. Growth was continued at 18 °C overnight. Bacteria were collected through centrifugation, and the obtained pellets were immediately used for the subsequent steps. The pellets were resuspended in lysis buffer (20 mM PB, 150 mM sodium chloride, pH 7.4) containing a protease inhibitor cocktail. Cell lysis was performed in an ultrasonic ice bath to generate crude protein samples. Cleaved protein samples were subsequently diluted fivefold using balance buffer (500 mM sodium chloride, 20 mM Tris, pH 8.0) and incubated with Ni-agarose beads (CWBIO) to remove uncleaved proteins and proteases. Proteins were eluted from the beads using different concentrations of imidazole (i.e., 20, 50, 200, and 500 mM), and the absorption peak was detected. Subsequent samples were eluted using the imidazole concentration indicated by the absorption peak.

### Gene expression data

Survival analysis of (publicly available) gene expression microarray data was done using GenomicScape online (http://genomicscape.com). The University of Arkansas for Medical Sciences (UAMS) TT2 and TT3 cohorts (dataset GSE4581; http:// www. ncbi.nlm.nih.gov/geo/query/acc.cgi?acc=GSE4581) contain the expression data of malignant plasma cells (PC) from the bone marrow of newly diagnosed, untreated MM patients.

### Signal-Seeker™ ubiquitination detection assay

RPMI-8266 cells were treated with BBR (25 μM) for 24 h and harvested. Cells were lysed on ice in 1× BlastR™ Lysis Buffer with de-ubiquitinase inhibitor (part. #NEM09BB, Cytoskeleton) and protease inhibitor cocktail (cat. #PIC02, Cytoskeleton). Ubiquitinated proteins were immunoprecipitated using Signal-Seeker™ Ubiquitination Detection Kit according to the protocol provided by the manufacturer (cat. #BK161, Cytoskeleton). Briefly, the appropriate amount of Ub (cat. #UBA01, Cytoskeleton) or control beads (Cat. #CUB01, Cytoskeleton) was added to the respective samples for 1–2 h at 4 °C on an end-over-end tumbler. Following incubation, the affinity beads from each sample were pelleted and washed thrice with Blast-R wash buffer. Bound proteins were eluted using the elution buffer and spin columns of the Signal-Seeker kits, and post-translational modified target proteins were detected through western blotting with anti-UHRF1 antibody.

### SPR-LC-MS/MS approach

Three-dimensional (3D) Photo-cross-linker Sensor CHIP™ used in this part was provided by Betterways Inc., China. This chip can immobilize BBR without chemical label linking. For spotting, a BioDot™ AD-1520 Array Printer (BIODOT Inc., USA) printed BBR and controls on the chip surface. The solvent in the sample dots was evaporated in a dark N2 atmosphere, and the sensor chips were quickly transferred to a UV spectroirradiator (Amersham Life Science, USA) for a photo-cross-linking reaction. Surface plasmon resonance (SPR) analysis was performed to validate the BBR-UHRF1 domain interaction, and the optimum resonance angle was automatically tuned using bScreen LB 991 Label-free Microarray System (Berthold technologies, Germany). For sequential binding assays, chips were pretreated with BBR or mock-treated (rapamycin was used as a positive binding control) with a single injection (5 mL/min for 1 min), and subsequently exposed to proteins. The retained resonance units were recorded, and triplicate values were averaged [43]. After the SPR test, the chip was collected and subjected to in situ enzymatic hydrolysis with trypsin, and the enriched protein on the chip surface was subsequently identified through HPLC-MS (Fitgene Biotechnology, China). Hemi-methylated DNA (12 bp, upper strand: 5′-GGGCCXGCAGGG-3′, lower strand: 5′-GCAGGCGGCCTC-3′, X = 5-methyldeoxycytosine) was synthesized by TsingKe Co., Ltd., Beijing, China. Purified UHRF1 protein was pre-incubated with hemi-methylated DNA at the indicated molar ratios (1:2) for 10 min at 4 °C; SPR analysis was performed.

### Molecular docking

The molecular structures of UHRF1 domains (PDB: 2FAZ, PDB: 4GY5, PDB: 2PB7, and PDB: 3FL2) were obtained from PDB. Amino acid gaps were automatically filled using the Home-building program. Molecular docking was performed using Maestro 9.0 Schrodinger program, following the standard procedures described in the manual of the software.

### Xenograft mouse model of MM cell lines

All animal experiments were approved by and conformed to the relevant regulatory standards of the Institutional Animal Care and Use Committee at the Institute of Laboratory Animal Science, Jinan University (Guangzhou, China). For the animal study, BALB/c nude mice (4 weeks old, female) were used for the MM experiments in vivo and maintained in a temperature- and humidity-controlled environment. A total of 2 **×** 10^7^ RPMI-8266 cells were subcutaneously injected into sub-lethally irradiated (3 Gy) BALB/c mice. When the volume of the tumors was measurable (i.e., 100–180 mm^3^), the mice were randomized into treatment groups and received 50 mg/kg BBR in 0.2 mL saline solution intragastrically for 2 days. The animals were monitored for tumor volume through caliper measurements every alternate day. Tumor volume was estimated using the following formula: (length) × (width)^2^/2. Animals were euthanized through CO_2_ inhalation in the event of a tumor size > 2 cm or tumor ulceration, in accordance with the guidelines established by the Jinan University Animal Care and Use Committee. Survival was evaluated from the first day of treatment until death.

### Statistical analysis

Statistical analysis was performed using GraphPad Prism 7 (GraphPad). The results are expressed as means ± standard deviation. The significance of differences among groups was evaluated through one-way analysis of variance with post hoc Bonferroni test. Paired analyses were performed using Student’s *t* test. *p < 0.05, **p < 0.01, and ***p < 0.001 denoted statistical significance. The Kaplan-Meier survival curves were analyzed using the log-rank test.

## Supplementary information

**Additional file 1: Figure S1.** The effect of BBR and bortezomib on the proteasome activity and protein ubiquitination level in MM cells.

**Additional file 2: Table S1.** Unique targets of BBR in RPMI-8266 cell lines.

**Additional file 3: Table S2.** Unique targets of BBR in MM.1S cell lines.

**Additional file 4: Table S3.** Common targets of BBR present in both RPMI-8266 and MM.1S cell lines.

**Additional file 5: Figure S2.** The UHRF1 protein expression in MM cell lines and nor hPBMCs.

**Additional file 6: Table S4.** The interaction of BBR-UHRF1 were detected by SPR analysis.

**Additional file 7: Figure S3.** Zoom-in view of the "PDNPKERGFWYD" peptide and BBR in the stick representation, labeled by residue name and position.

**Additional file 8: Figure S4.** Proteins were purified from *E. coli* lysates overexpressing different domains of UHRF1.

**Additional file 9: Figure S5.** The effect of BBR on UHRF1 R235A and UHRF1 protein expression.

**Additional file 10: Figure S6.** MM.1S and RPMI-8266 cells were treated with lysosome inhibitor (chloroquine 100 μM), autophagy inhibitor (3-MA, 25 μM) or BBR (25 μM) for indicated time. Cells lysates were harvested and subjected to western blotting with the anti-UHRF1 and anti-GAPDH antibodies.

**Additional file 11: Figure S7.** The stable MM cell lines with transfected control vector and lentiviral-UHRF1 were established and cell lysates were subjected to western blotting with the anti-UHRF1 and anti-GAPDH antibodies.

**Additional file 12: Table S5.** Antibodies.

**Additional file 13: Table S6.** Experimental models.

**Additional file 14: Table S7.** Chemicals, recombinant proteins, and plasmids.

**Additional file 15: Table S8.** Oligonucleotides.

## Data Availability

Materials are available upon reasonable request. The dataset used in this study is available through the following means: Dataset GSE4581 was available in http://www.ncbi.nlm.nih.gov/geo/query/acc.cgi?acc=GSE4581 and was sourced from Shaughnessy Jr. John [44]. Survival analysis was performed in GenomicScape (http://genomicscape.com).

## Abbreviations

BBR: Berberine
MM: Multiple myeloma
UHRF1: Ubiquitin-like, with PHD and RING finger domains 1
IL-6: Interleukin-6
SPR-LC-MS/MS: Surface plasmon resonance-liquid chromatography-tandem mass spectrometery system
SCF-β-TrCP: The Skp, Cullin, F-box containing complex-β-Transducin Repeat Containing Protein Complex
DNMT1: DNA methyltransferase 1
BM: Bone marrow
IC50: The half maximal inhibitory concentration
PBMCs: Peripheral blood mononuclear cells
BTZ: Bortezomib
PSM: Peptide-Spectrum Match
Kd: Dissociation constant
TSGs: Tumor suppressor genes
