# Supplementary material for: Identification of berberine as a novel drug for the treatment of multiple myeloma via targeting UHRF1
Source: BMC Biol. 2020 Mar 25;18:33. doi: 10.1186/s12915-020-00766-8 (PMC7098108; doi:10.1186/s12915-020-00766-8)
Supplement: Supplementary file 1 — Additional file 1: Figure S1. The effect of BBR and bortezomib on the proteasome activity and protein ubiquitination level in MM cells. [file 12915_2020_766_MOESM1_ESM.pdf]

Additional file 1, Figure S1.

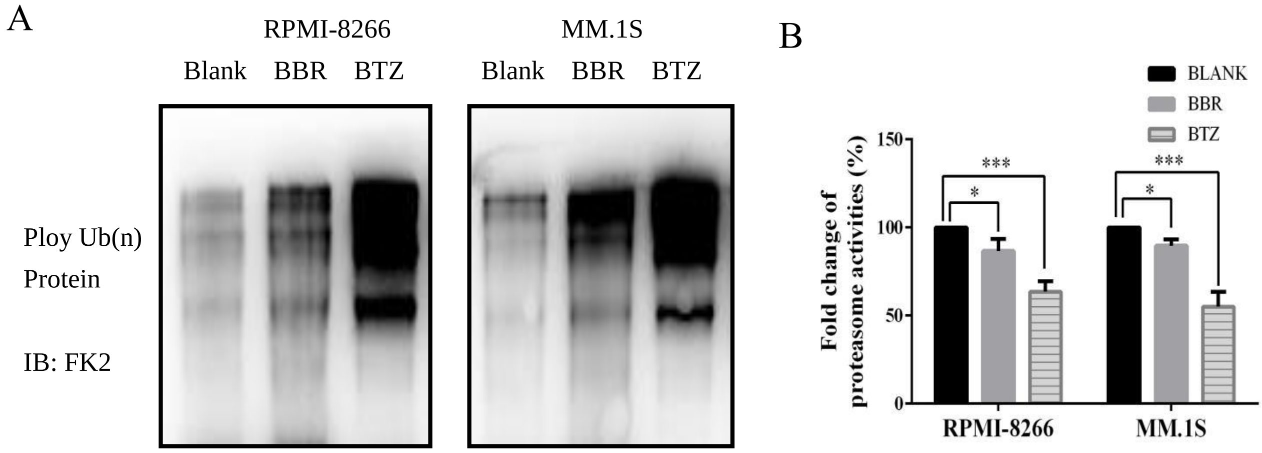

Additional file 1, Figure S1. The effect of BBR and bortezomib on the proteasome activity and protein ubiquitination level in MM cells.

A. RPMI-8266 and MM.1S cells were treated with DMSO, BBR, or BTZ for 3h, and protein lysates were analyzed for proteasome activity. Protein lysates from control, BBR- or BTZ-treated RPMI-8266, and MM.1S cells were subjected to IB with an anti-FK2 antibody.

B. Raw data were normalized to percent proteasome activity in control (as 100%) versus drug-treated cells (mean ± SD; n=3; *, $P < 0.05$; **, $P < 0.01$； ***, $P < 0.001$ compared with BLANK).
