# Supplementary material for: Identification of berberine as a novel drug for the treatment of multiple myeloma via targeting UHRF1
Source: BMC Biol. 2020 Mar 25;18:33. doi: 10.1186/s12915-020-00766-8 (PMC7098108; doi:10.1186/s12915-020-00766-8)
Supplement: Supplementary file 2 — Additional file 2: Table S1. Unique targets of BBR in RPMI-8266 cell lines. [file 12915_2020_766_MOESM2_ESM.pdf]

Additional file 2, Table S1. Unique targets of BBR in RPMI-8266 cell lines.

Entry	Score_RPMI-8266	PSMs_RPMI-8266
O14757_CHEK1	1675.86	78
P02533_KRT14	12.11	1
P11274_BCR	1195.72	12
P20042_EIF2S2	121.87	1
P21333_FLNA	148.25	7
P68032_ACTC1	61.09	1
