# Supplementary material for: Identification of berberine as a novel drug for the treatment of multiple myeloma via targeting UHRF1
Source: BMC Biol. 2020 Mar 25;18:33. doi: 10.1186/s12915-020-00766-8 (PMC7098108; doi:10.1186/s12915-020-00766-8)
Supplement: Supplementary file 3 — Additional file 3: Table S2. Unique targets of BBR in MM.1S cell lines. [file 12915_2020_766_MOESM3_ESM.pdf]

Additional file 3, Table S2. Unique targets of BBR in MM.1S cell lines.

Entry	Score_MM.1S	PSMs_MM.1S
O95644_NFAC1	668.29	20
P05023_AT1A1	846.24	48
P06213_INSR	653.29	39
P20020_AT2B1	322.91	10
P28482_MK01	761.82	11
Q08209_PP2BA	850.96	19
Q9Y243_AKT3	342.01	6
