# Supplementary material for: Identification of berberine as a novel drug for the treatment of multiple myeloma via targeting UHRF1
Source: BMC Biol. 2020 Mar 25;18:33. doi: 10.1186/s12915-020-00766-8 (PMC7098108; doi:10.1186/s12915-020-00766-8)
Supplement: Supplementary file 4 — Additional file 4: Table S3. Common targets of BBR present in both RPMI-8266 and MM.1S cell lines. [file 12915_2020_766_MOESM4_ESM.pdf]

Additional file 4, Table S3. Common targets of BBR present in both RPMI-8266 and MM.1S cell lines.

Entry	Score_RPMI-8266	Score_MM.1S	PSMs_RPMI-8266	PSMs_MM.1S
Q16665_HIF1A	1678.03	2124.42	91	81
P19875_CXCL2	1690.88	2007.29	4	5
O43613_HCRTR1	1669.58	2002.05	32	42
O60674_JAK2	1864.45	1988.57	152	90
P53539_FOSB	1798.11	1616.37	21	27
P68371_TUBB4B	1454.92	1595.09	9	6
P22303_ACHE	1752.35	1594.27	13	8
P10828_THRB	1288.68	1583.71	5	8
Q15722_LTB4R	1322.54	1528.13	23	30
P10636_MAPT	1368.62	1504.12	15	18
P11245_NAT2	1657.77	1498.43	2	2
P13726_F3	1456.92	1477.26	25	19
P10275_AR	1609.32	1465.06	30	39
P62072_TIMM10	1559.41	1461.78	4	3
P11229_CHRM1	1166.17	1421.48	25	26
P10635_CYP2D6	1418.64	1411.08	13	16
P11387_TOP1	1276.01	1372.55	66	92
O15392_BIRC5	1345.01	1350.81	21	17
P37231_PPARG	1281.51	1323.64	30	32
Q13509_TUBB3	1441.07	1321.60	33	23
Q96LD8_SENP8	1369.41	1321.56	44	52
Q99720_SIGMAR1	1159.10	1316.35	16	17
P35869_AHR	1468.80	1284.93	66	61
P25101_EDNRA	1012.79	1246.44	5	5
Q9Y6K9_IKBKG	1202.26	1134.33	39	37
P24468_NR2F2	997.58	1102.92	5	5
Q96T88_UHRF1	996.09	1099.87	101	66
P05231_IL6	1010.41	1070.65	1	2
P08684_CYP3A4	955.61	1046.09	30	37
Q71U36_TUBA1A	943.86	1040.84	10	11
P05362_ICAM1	897.19	1031.80	9	10
P03372_ESR1	880.79	1007.13	24	17
Q13639_HTR4	1102.88	964.71	27	26
P40763_STAT3	981.01	915.90	34	36
P07437_TUBB	757.45	840.55	8	7
P35228_NOS2	832.44	838.99	12	9
P06276_BCHE	725.09	823.76	18	20
P35354_PTGS2	874.38	765.75	5	4

P08151_GLI1	686.18	761.80	49	47
P24530_EDNRB	635.95	745.18	5	6
P18089_ADRA2B	716.88	708.45	6	8
Q16236_NFE2L2	766.20	696.05	28	40
P00519_ABL1	765.24	670.62	45	43
P04406_GAPDH	558.66	645.18	10	8
P05412_JUN	678.47	638.36	1	2
P04637_TP53	698.38	574.02	11	8
Q92698_RAD54L	492.26	559.68	22	27
Q07820_MCL1	504.43	514.56	1	1
P41595_HTR2B	505.67	489.06	9	11
Q02880_TOP2B	590.68	487.01	23	20
P16581_SELE	470.07	486.93	13	16
P13500_CCL2	569.88	482.18	1	2
Q14289_PTK2B	503.98	482.07	10	14
P43351_RAD52	469.64	475.24	5	4
P10415_BCL2	491.42	391.82	7	7
Q9NUW8_TDP1	410.33	359.19	1	1
P24385_CCND1	338.76	328.95	1	1
P29460_IL12B	306.83	307.10	1	1
P62263_RPS14	233.01	295.86	1	1
P68363_TUBA1B	256.89	277.67	1	2
P50454_SERPINH1	254.11	246.36	1	1
O43143_DHX15	182.83	233.65	4	4
P62701_RPS4X	231.81	209.39	1	1
P43243_MATR3	206.20	191.46	6	6
Q14566_MCM6	220.28	183.17	4	4
P31327_CPS1	156.54	172.67	3	3
P21796_VDAC1	163.77	167.00	1	2
Q02878_RPL6	153.62	132.98	1	1
P61353_RPL27	153.54	124.30	1	1
P21291_CSRP1	136.85	124.21	1	1
P02538_KRT6A	101.52	86.99	1	2
P49368_CCT3	80.95	86.04	1	2
P61313_RPL15	75.68	84.21	1	2
P46013_MKI67	95.19	77.16	1	1
P60709_ACTB	60.79	61.41	1	2
P00338_LDHA	60.47	57.58	1	1
P11586_MTHFD1	52.97	43.29	1	1
P22626_HNRNPA2B1	42.38	36.28	1	2
Q9BTV4_TMEM43	26.48	21.58	1	1
P23246_SFPQ	19.48	19.31	1	2
Q9Y2X3_NOP58	5.52	5.60	1	2
