# Supplementary material for: Identification of berberine as a novel drug for the treatment of multiple myeloma via targeting UHRF1
Source: BMC Biol. 2020 Mar 25;18:33. doi: 10.1186/s12915-020-00766-8 (PMC7098108; doi:10.1186/s12915-020-00766-8)
Supplement: Supplementary file 5 — Additional file 5: Figure S2. The UHRF1 protein expression in MM cell lines and nor hPBMCs. [file 12915_2020_766_MOESM5_ESM.pdf]

Additional file 5, Figure S2

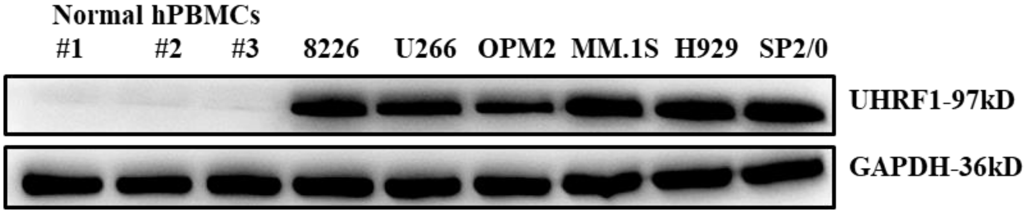

Additional file 5, Figure S2. The UHRF1 protein expression in MM cell lines and normal hPBMCs. Cell lysates MM cells and normal hPBMCs lysates were subjected to western blotting with anti-UHRF1 and anti-GAPDH antibodies. The protein levels of UHRF1 were higher in MM cells than normal hPBMCs.
