# Supplementary material for: Identification of berberine as a novel drug for the treatment of multiple myeloma via targeting UHRF1
Source: BMC Biol. 2020 Mar 25;18:33. doi: 10.1186/s12915-020-00766-8 (PMC7098108; doi:10.1186/s12915-020-00766-8)
Supplement: Supplementary file 6 — Additional file 6: Table S4. The interaction of BBR-UHRF1 were detected by SPR analysis. [file 12915_2020_766_MOESM6_ESM.pdf]

Additional file 6, Table S4. The interaction of BBR-UHRF1 were detected by SPR analysis.

NO.	stationary phase	mobile phase	Avg K_{ON} (1/MS)	Avg K_{OFF} (1/S)	Avg KD (M)	interactions intensity
1	BBR	Pep-1	1.75E+00	4.44E-01	2.54E-01	VW/None
2	BBR	Pep-2	1.65E+00	1.66E-01	1.01E-01	VW/None
3	BBR	Pep-3	1.20E+02	4.40E-02	3.68E-04	Middle
4	BBR	UHRF1	2.93E+03	3.81E-03	1.30E-06	Stronge
5	BBR	UHRF1 R235A	2.96E+02	2.84E-01	9.61E-04	Middle
6	BBR	UHRF1 + hm-DNA	8.75E+03	4.03E-03	4.60E-07	Strong
7	BBR	hm-DNA	1.75E+00	6.03E-01	3.45E-01	VW/None
8	BBR	TTD-PHD	3.64E+03	1.08E-02	2.96E-06	Stronge
9	BBR	TTD-PHD R235A	1.79E+02	4.02E-01	2.25E-03	Weak
10	BBR	NRIF	2.94E+01	3.68E-01	1.25E-02	Weak
12	BBR	PHD	1.12E+01	1.28E-01	1.14E-02	Weak
13	BBR	SRA	4.15E+00	7.87E-02	1.90E-02	Weak
14	BBR	RING	1.56E+01	8.33E-02	5.34E-03	Weak
16	BBR	PBS	1.13E+00	6.54E-01	5.80E-01	VW/None
