# Supplementary material for: Identification of berberine as a novel drug for the treatment of multiple myeloma via targeting UHRF1
Source: BMC Biol. 2020 Mar 25;18:33. doi: 10.1186/s12915-020-00766-8 (PMC7098108; doi:10.1186/s12915-020-00766-8)
Supplement: Supplementary file 7 — Additional file 7: Figure S3. Zoom-in view of the "PDNPKERGFWYD" peptide and BBR in the stick representation, labeled by residue name and position. [file 12915_2020_766_MOESM7_ESM.pdf]

Additional file 7, Figure S3

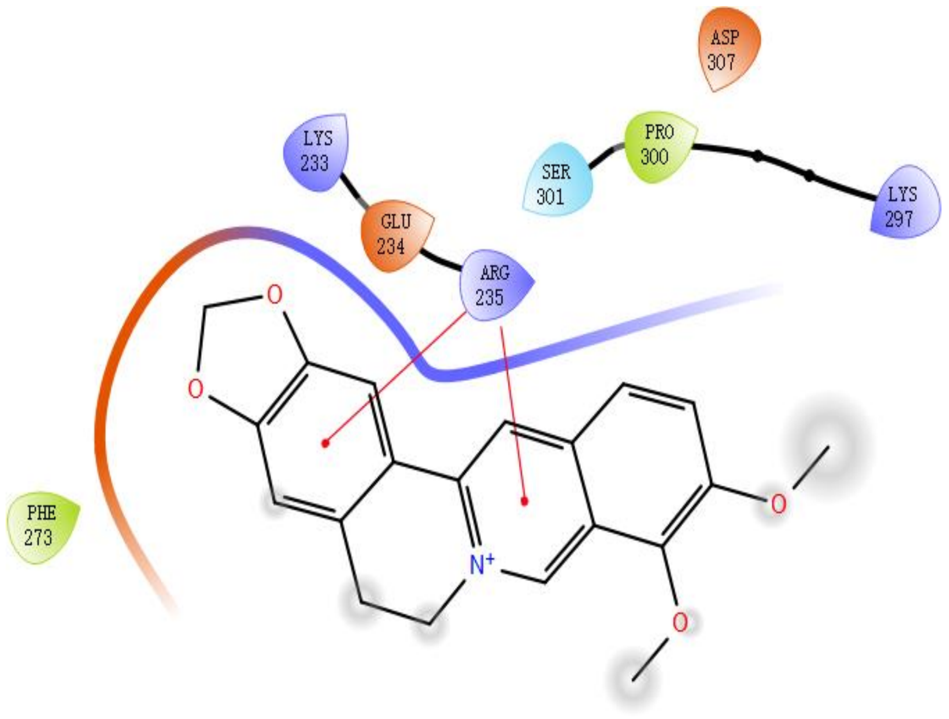

Additional file 7, Figure S3. Zoom-in view of the "229-PDNPKERGFWYD-240" peptide and BBR in the stick representation, labeled by residue name and position.
