# Supplementary material for: Identification of berberine as a novel drug for the treatment of multiple myeloma via targeting UHRF1
Source: BMC Biol. 2020 Mar 25;18:33. doi: 10.1186/s12915-020-00766-8 (PMC7098108; doi:10.1186/s12915-020-00766-8)
Supplement: Supplementary file 8 — Additional file 8: Figure S4. Proteins were purified from E. coli lysates overexpressing different domains of UHRF1. [file 12915_2020_766_MOESM8_ESM.pdf]

Additional file 8, Figure S4.

A UHRF1

1 2 3 4 5 6

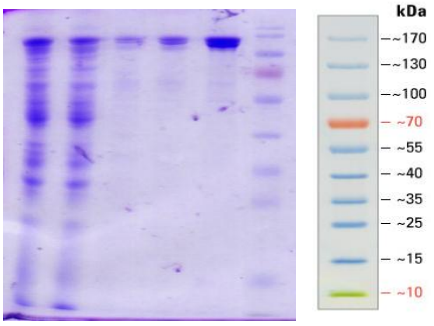

Lane 1: Supernatant
Lane 2: Flow through
Lane 3: Elution by 20mM imidazole
Lane 4: Elution by 50mM imidazole
Lane 5: Elution by 250mM imidazole
Lane 6: Marker

B UHRF1-R235A

1 2 3 4 5 6

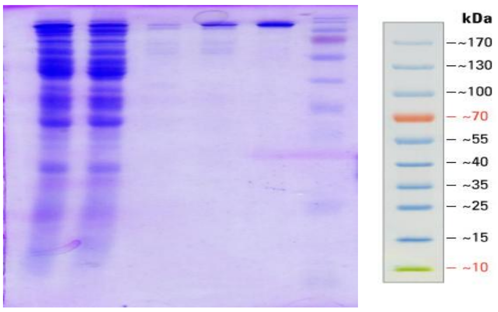

Lane 1: Supernatant
Lane 2: Flow through
Lane 3: Elution by 20mM imidazole
Lane 4: Elution by 50mM imidazole
Lane 5: Elution by 250mM imidazole
Lane 6: Marker

C TTD-PHD

1 2 3 4 5 6 7

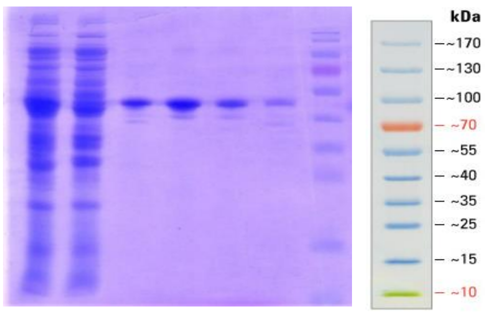

Lane 1: Supernatant
Lane 2: Flow through
Lane 3: Elution by 20mM imidazole
Lane 4: Elution by 50mM imidazole
Lane 5: Elution by 250mM imidazole
Lane 6: Beads
Lane 7: Marker

D TTD-PHD-R235A

1 2 3 4 5 6 7

Lane 1: Marker
Lane 2: Supernatant
Lane 3: Flow through
Lane 4: Elution by 20mM imidazole
Lane 5: Elution by 50mM imidazole
Lane 6: Elution by 250mM imidazole

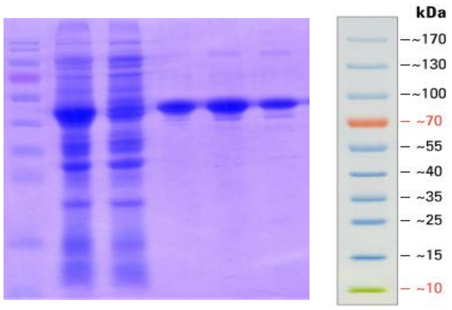

E NIRF 1 2 3 4 5 6

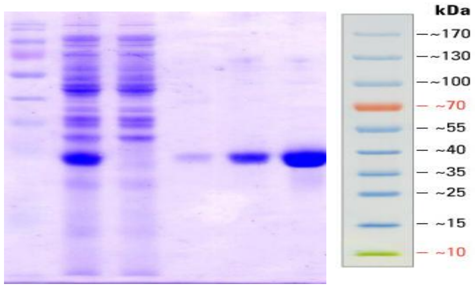

Lane1: Marker
Lane2: Supernatant
Lane3: Flow through
Lane4: Elution by 20mM imidazole
Lane5: Elution by 50mM imidazole
Lane6: Elution by 250mM imidazole

F PHD 1 2 3 4 5 6

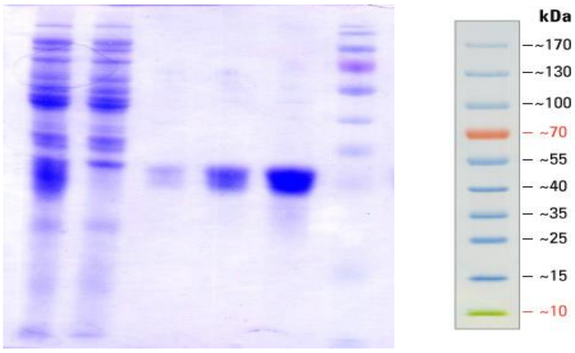

Lane 1: Supernatant
Lane 2: Flow through
Lane 3: Elution by 20mM imidazole
Lane 4: Elution by 50mM imidazole
Lane 5: Elution by 250mM imidazole
Lane 6: Marker

G SRA and RING 1 2 3 4 5 6 7 8 9 10 11

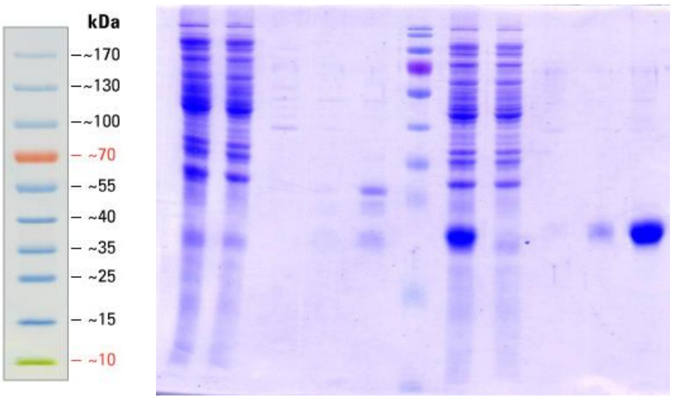

Lane1: SRA-Supernatant
Lane2: SRA-Flow through
Lane3: SRA-Elution by 20mM imidazole
Lane4: SRA-Elution by 50mM imidazole
Lane5: SRA-Elution by 250mM imidazole
Lane6: Marker
Lane7: RING-Supernatant
Lane8: RING-Flow through
Lane9: RING-Elution by 20mM imidazole
Lane10: RING-Elution by 50mM imidazole
Lane11: RING-Elution by 250mM imidazole

Additional file 8, Figure S4. Proteins were purified from *E. coli* lysates overexpressing different domains of UHRF1.

A. UHRF1. B. UHRF1-R235A. C. TTD-PHD. D. TTD-PHD-R235A. E. NIRF. F. PHD. G. SRA and RING.
