# Supplementary material for: Identification of berberine as a novel drug for the treatment of multiple myeloma via targeting UHRF1
Source: BMC Biol. 2020 Mar 25;18:33. doi: 10.1186/s12915-020-00766-8 (PMC7098108; doi:10.1186/s12915-020-00766-8)
Supplement: Supplementary file 9 — Additional file 9: Figure S5. The effect of BBR on UHRF1 R235A and UHRF1 protein expression. [file 12915_2020_766_MOESM9_ESM.pdf]

Additional file 9, Figure S5

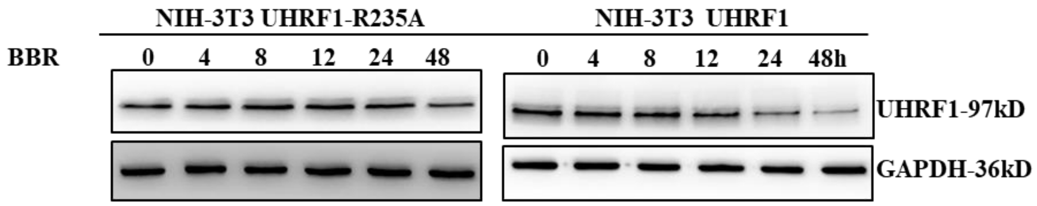

Additional file 9, Figure S5. The effect of BBR on UHRF1 R235A and UHRF1 protein expression. pFlag-CMV4-UHRF1 and pFlag-CMV4-UHRF1 R235A were transfected into NIH 3T3 cells, and treated with 25 μM BBR for 0, 4, 8, 12, 24, and 48 h. Cells lysates were harvested and subjected to western blotting with the anti-flag and anti-GAPDH antibodies.
