# Supplementary material for: Identification of berberine as a novel drug for the treatment of multiple myeloma via targeting UHRF1
Source: BMC Biol. 2020 Mar 25;18:33. doi: 10.1186/s12915-020-00766-8 (PMC7098108; doi:10.1186/s12915-020-00766-8)
Supplement: Supplementary file 10 — Additional file 10: Figure S6. MM.1S and RPMI-8266 cells were treated with lysosome inhibitor (chloroquine 100 μM), autophagy inhibitor (3-MA, 25 μM) or BBR (25 μM) for indicated time. Cells lysates were harvested and subjected to western blotting with the anti-UHRF1 and anti-GAPDH antibodies. [file 12915_2020_766_MOESM10_ESM.pdf]

Additional file 10, Figure S6

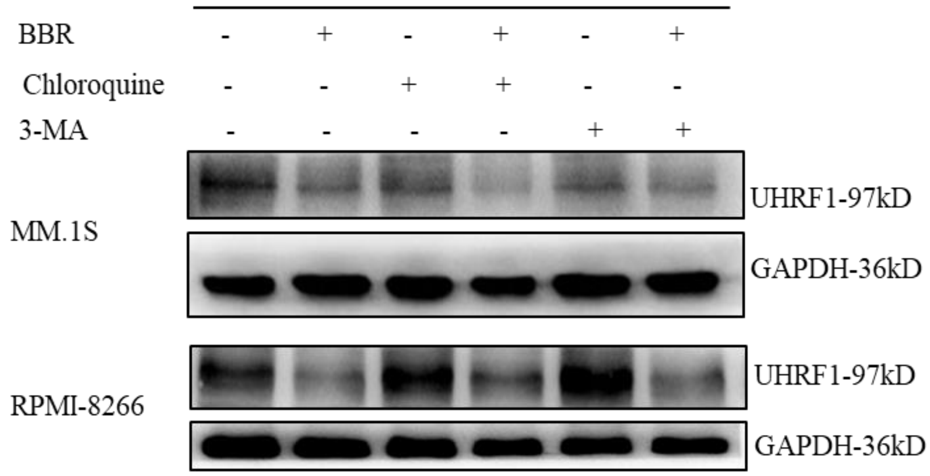

Additional file 10, Figure S6. MM.1S and RPMI-8266 cells were treated with lysosome inhibitor (chloroquine 100 μM), autophagy inhibitor (3-MA, 25μM) or BBR (25 μM) for indicated time. Cells lysates were harvested and subjected to western blotting with the anti-UHRF1 and anti-GAPDH antibodies.
