# Supplementary material for: Identification of berberine as a novel drug for the treatment of multiple myeloma via targeting UHRF1
Source: BMC Biol. 2020 Mar 25;18:33. doi: 10.1186/s12915-020-00766-8 (PMC7098108; doi:10.1186/s12915-020-00766-8)
Supplement: Supplementary file 11 — Additional file 11: Figure S7. The stable MM cell lines with transfected control vector and lentiviral-UHRF1 were established and cell lysates were subjected to western blotting with the anti-UHRF1 and anti-GAPDH antibodies. [file 12915_2020_766_MOESM11_ESM.pdf]

Additional file 11, Figure S7

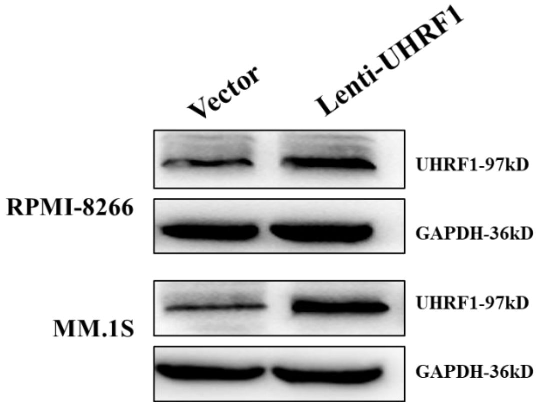

Additional file 11, Figure S7. The stable MM cell lines with transfected control vector and lentiviral-UHRF1 were established and cell lysates were subjected to western blotting with the anti-UHRF1 and anti-GAPDH antibodies.
