# Supplementary material for: Identification of berberine as a novel drug for the treatment of multiple myeloma via targeting UHRF1
Source: BMC Biol. 2020 Mar 25;18:33. doi: 10.1186/s12915-020-00766-8 (PMC7098108; doi:10.1186/s12915-020-00766-8)
Supplement: Supplementary file 12 — Additional file 12: Table S5. Antibodies. [file 12915_2020_766_MOESM12_ESM.pdf]

Additional file 12, Table S5. Antibodies.

Reagent or Resource	Supplier	Identifier
Anit-UHRF1	Abcam	Cat# ab213223
Anti-DNMT1	Cell Signaling Technology	Cat# 5032
Anti-Ubiquitin (fk2)	Millipore	Cat# ST1200
Anti-p53	Cell Signaling Technology	Cat# 2524
Anti-p16INK4A	Cell Signaling Technology	Cat# 80772S
Anti-p73	Cell Signaling Technology	Cat# 14620
Anti-rabbit IgG, HRP-linked	Cell Signaling Technology	Cat# 7074S
Anti-rabbit IgG, HRP-linked	Cell Signaling Technology	Cat# 7076S
Anti-GAPDH	Cell Signaling Technology	Cat# 2118
