# Supplementary material for: Identification of berberine as a novel drug for the treatment of multiple myeloma via targeting UHRF1
Source: BMC Biol. 2020 Mar 25;18:33. doi: 10.1186/s12915-020-00766-8 (PMC7098108; doi:10.1186/s12915-020-00766-8)
Supplement: Supplementary file 13 — Additional file 13: Table S6. Experimental models. [file 12915_2020_766_MOESM13_ESM.pdf]

Additional file 13, Table S6. Experimental models

Experimental model	Source
BALB/c nu: female;	Institute of Laboratory Animal Science, Jinan University
C57BL/6J	Institute of Laboratory Animal Science, Jinan University
