# Supplementary material for: Identification of berberine as a novel drug for the treatment of multiple myeloma via targeting UHRF1
Source: BMC Biol. 2020 Mar 25;18:33. doi: 10.1186/s12915-020-00766-8 (PMC7098108; doi:10.1186/s12915-020-00766-8)
Supplement: Supplementary file 14 — Additional file 14: Table S7. Chemicals, recombinant proteins, and plasmids. [file 12915_2020_766_MOESM14_ESM.pdf]

Additional file 14, Table S7. Chemicals, recombinant proteins, and plasmids.

Chemicals, recombinant proteins, and plasmids	Source	Identifier
Berberine	Sigma–Aldrich	S1026
Chloroquine	Sigma–Aldrich	C6628
3-MA	Sigma–Aldrich	M9281
MG-132	Sigma–Aldrich	M7449
Cycloheximide	MedChem Express	HY-12320
Bortezomib	MedChem Express	HY-10227
pCMV3-N-Flag-UHRF1	Sino Biological	HG17896-NF
pRK-5-N-HA-Ubiquitin	Miaolinbio	P1761
LPP-CS-UHRF1-Lv130-01-400	GeneCopoeia	N/A
LPP-CS-NEG-Lv130-100	GeneCopoeia	N/A
Protein A/G PLUS-Agarose	Santa Cruz Biotechnology	Sc-2003
Anti-Flag Affinity Gel	Bimake	B23101
Signal-SeekerTM Ubiquitination Detection Kit	Cytoskeleton	BK161
Lymphoprep™	Stem cell	#07851
20S Proteasome Activity Assay Kit	Millipore	Cat. No. APT280
