# Supplementary material for: Identification of berberine as a novel drug for the treatment of multiple myeloma via targeting UHRF1
Source: BMC Biol. 2020 Mar 25;18:33. doi: 10.1186/s12915-020-00766-8 (PMC7098108; doi:10.1186/s12915-020-00766-8)
Supplement: Supplementary file 15 — Additional file 15: Table S8. Oligonucleotides. [file 12915_2020_766_MOESM15_ESM.pdf]

Additional file 15, Table S8. Oligonucleotides.

| Oligonucleotide | Sequence(5′ to 3′) | Source |
| --- | --- | --- |
| UHRF1-siRNA #1 | | Guangzhou RiboBio Co., Ltd. |
| Sense | CGAGAGCGAGAGAAGGAGAACAGCA tt | |
| Antisense | UGCUGUUCUCCUUCUCUCGCUCUCG tt | |
| UHRF1-siRNA #2 | | Guangzhou RiboBio Co., Ltd. |
| Sense | GAGAGCGAGAGAAGGAGAACAGCAA tt | |
| Antisense | UUGCUGUUCUCCUUCUCUCGCUCUC tt | |
| UHRF1-siRNA #3 | | Guangzhou RiboBio Co., Ltd. |
| Sense | GAGCGAGAGAAGGAGAACAGCAAGA tt | |
| Antisense | UCUUGCUGUUCUCCUUCUCUCGCUC tt | |
| Negative Control siRNA | | Guangzhou RiboBio Co., Ltd. |
| Sense | GAGAGAAAGAGGAAGGACACCGAGA tt | |
| Antisense | UCUCGGUGUCCUUCCUCUUUCUCUC tt | |
| GAPDH Primers | | Sangon Biotech (Shanghai) Co., Ltd. |
| Forward | CAACGGATTTGGTCGTATT | |
| Reverse | CACAGTCTTCTGGGTGGC | |
| UHRF1 Primers | | Sangon Biotech (Shanghai) Co., Ltd. |
| Forward | GGCAAGTGGAAGCGGAAGTCG | |
| Reverse | CTTGGCGTTGCTCTTGTCCTCTC | |
| P16INK4A Primers | | Sangon Biotech (Shanghai) Co., Ltd. |
| Forward | TCATGATGATGGGCAGCG | |

| | | |
|---|---|---|
| Reverse | CATCTATGCGGGCATGGTTA | |
| P53 Primers | | Sangon Biotech (Shanghai) Co., Ltd. |
| Forward | CCAACAACACCAGCTCCTCT | |
| Reverse | CAAGGCCTCATTCAGCTCTC | |
| P73 Primers | | Sangon Biotech (Shanghai) Co., Ltd. |
| Forward | CCTTGGTGCCGGTGTGAAGAAG | |
| Reverse | GCTGCTGCTGCTGCCGATAG | |
